# *In vivo* Phage Display: A promising selection strategy for the improvement of antibody targeting and drug delivery properties

**DOI:** 10.3389/fmicb.2022.962124

**Published:** 2022-09-26

**Authors:** Ana S. André, Isa Moutinho, Joana N. R. Dias, Frederico Aires-da-Silva

**Affiliations:** ^1^Centro de Investigação Interdisciplinar em Sanidade Animal (CIISA), Faculdade de Medicina Veterinária, Universidade de Lisboa, Avenida da Universidade Técnica, Lisbon, Portugal; ^2^Laboratório Associado para Ciência Animal e Veterinária (AL4AnimalS), Lisbon, Portugal

**Keywords:** phage display, *in vivo*, antibody discovery, antibody engineering, antibody selection, therapeutic antibodies

## Abstract

The discovery of hybridoma technology, described by Kohler and Milstein in 1975, and the resulting ability to generate monoclonal antibodies (mAbs) initiated a new era in antibody research and clinical development. However, limitations of the hybridoma technology as a routine antibody generation method in conjunction with high immunogenicity responses have led to the development of alternative approaches for the streamlined identification of most effective antibodies. Within this context, display selection technologies such as phage display, ribosome display, yeast display, bacterial display, and mammalian cell surface display have been widely promoted over the past three decades as ideal alternatives to traditional hybridoma methods. The display of antibodies on phages is probably the most widespread and powerful of these methods and, since its invention in late 1980s, significant technological advancements in the design, construction, and selection of antibody libraries have been made, and several fully human antibodies generated by phage display are currently approved or in various clinical development stages. With evolving novel disease targets and the emerging of a new generation of therapeutic antibodies, such as bispecific antibodies, antibody drug conjugates (ADCs), and chimeric antigen receptor T (CAR**-**T) cell therapies, it is clear that phage display is expected to continue to play a central role in antibody development. Nevertheless, for non-standard and more demanding cases aiming to generate best-in-class therapeutic antibodies against challenging targets and unmet medical needs, *in vivo* phage display selections by which phage libraries are directly injected into animals or humans for isolating and identifying the phages bound to specific tissues offer an advantage over conventional *in vitro* phage display screening procedures. Thus, in the present review, we will first summarize a general overview of the antibody therapeutic market, the different types of antibody fragments, and novel engineered variants that have already been explored. Then, we will discuss the state-of-the-art of *in vivo* phage display methodologies as a promising emerging selection strategy for improvement antibody targeting and drug delivery properties.

## Introduction

The discovery of hybridoma technology, described by Köhler and Milstein (1975), and the subsequent ability to develop monoclonal antibodies (mAbs) initiated a paradigm shift in antibody research and their clinical development. Yet, despite representing a major breakthrough in antibody-based therapy, early clinical studies demonstrated that unmodified murine mAbs presented properties that limited their use in the clinical setting. One of the most important shortcomings was the high immunogenic character of these mAbs that resulted in the generation of human anti-mouse antibody response (HAMA) that limited their therapeutic utility. Furthermore, murine mAbs demonstrated decreased serum half-life and inability to elicit human effector responses (Khazaeli et al., 1994; Hwang and Foote, 2005; Presta, 2006). To overcome these limitations, antibody engineering techniques have been explored and used to manipulate murine mAbs into chimeric or humanized antibodies by modifying their constant regions in human variants, which led to a reduction in HAMA response while promoting an efficacy optimization. More recently, fully human mAbs have being developed using hybridoma technology in transgenic mice models that have integrated into their germline human immunoglobulin (Ig) loci, such as HuMabMouse and XenoMouse platforms (Aires da Silva et al., 2008). Despite all these advances in antibody engineering and transgenic mice methods, the phage display technology has been considered, since its invention in the late 1980s, the most powerful technique for antibody discovery and development. By representing a robust and reliable method to identify specific high-affinity antigen binders from large combinatorial libraries of potentially clinically relevant antibodies, phage display technology has played a key role in the remarkable progress of discovering and optimizing antibodies for diverse applications, particularly antibody-based drugs. Nowadays, there are several antibodies and peptides generated by *in vitro* phage display currently approved or in advanced clinical stages. However, for more demanding cases aiming to generate best-in-class molecules against difficult targets, conventional *in vitro* approaches are not sufficient to fulfill unmet needs. Thus, *in vivo* phage display offers a technology capable of surpassing the drawbacks of the *in vitro* methods, making it a valuable tool to screen increasingly specific molecules. The current review provides a general overview of the antibody therapeutic market, the different types of antibody fragments, and engineered variants that have already been explored. Furthermore, we summarize the history and development of different types of antibody libraries and methods of selection with special focus on the phage display, particularly *in vivo.* Lastly, we review what has been achieved using this methodology for antibody fragments as well peptide libraries and then discuss how this strategy can improve the development of best-in-class monoclonal antibodies for cancer and other diseases.

## Monoclonal antibodies

The unique specificity and high efficacy of mAbs have made them effective molecules for therapeutic and diagnostic applications. Since the approval of Orthoclone OKT3®, the first monoclonal antibody approved by Emmons and Hunsicker (1987), the use of mAbs has become a new way to target antigens in a wide variety of diseases and conditions. Besides cancer and autoimmune disorders, mAbs are being used to treat over 50 other major diseases. Applications include treatment for heart disease, allergic conditions such as asthma, and prevention of organ rejection after transplantation. Mabs are also under investigation for the treatment of central nervous disorders such as Alzheimer’s disease, metabolic diseases like diabetes, and the prevention of migraines. Importantly, as medicine evolves into a new era of personalized therapy, the use of mAbs is at the core of this forefront. In fact, mAbs continue to be developed for newly identified biological targets and can be implemented into different formats to enhance their functionality and use, paving the way toward tailored medicines to each patient needs. Based on their commercial success and clinical potential, mAbs are among the top-selling drugs globally and their market size continues to grow annually. Presently, there are over 100 mAb products on the market; however, considering the thousands of candidates that are under preclinical and clinical trials and the novel advances in this field, this number is expected to increase substantially in the near future. The global monoclonal antibody market is estimated to generate the revenue of $300 billion by the end of 2025. In addition to their therapeutic applicability, the diagnosis is another area of potential expansion for these molecules. The global market for antibodies applied in diagnostics was valued at US$ 20,000 in 2017 and is projected to reach US$ 35,000 by 2026 at a CAGR of 5% from 2018 to 2026 (Parray et al., 2020).

## Antibody structure and function

**Figure 1:**
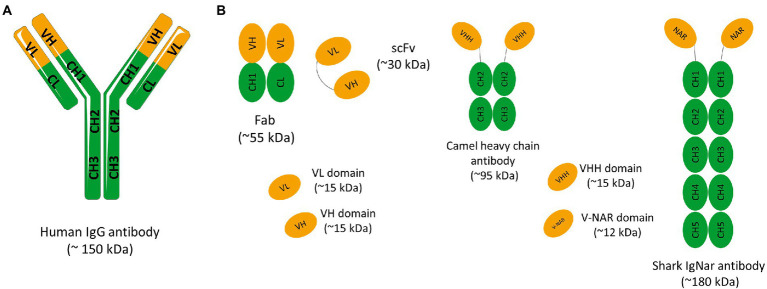
**(A)** Schematic representation of a conventional IgG antibody. Immunoglobulin G (IgG) have approximately 150 kDa and consist of two identical light chains (L) and two identical heavy chains (H) connected by disulfide bonds. Light chains are made up of a variable domain (VL) and a constant domain (CL). Heavy chains are made up of a variable domain (VH) and three constant domains (CH_1_, CH_2_ e CH_3_). The IgG molecule can also be divided into two main fragments: the antigen-binding-domain (Fab) and the fragment crystallizable (Fc) domain. The Fab fragment consists of two constant domains (CH_1_ and CL) and two variable domains (VH and VL). **(B)** Schematic representation of different antibody fragments. Fragment of antigen binding (Fab) composed of VL and a constant domain of the light chain (CL) linked to VH and a constant domain of the heavy chain (CH_1_) by a disulfide bond between the CL and CH_1_ domains. Single chain fragment variable (scFv) composed only of variable regions, one from the heavy chain (VH) and the other from the light chain (VL). The two variable regions are linked by a flexible glycine-serine linker (Gly_4_Ser)_3_. Camelid and shark immunoglobulin composed of only heavy chains. They present no light chain, and the displayed V domains bind their targets separately. Camelid heavy-chain antibodies composed a homodimer of one variable domain (VHH) and two C-like constant domains (CH). Shark new antigen receptor antibodies (IgNARs) composed of one variable domain (V-NAR) and five C-like constant domains (CH).

Antibodies, also named as immunoglobulins (Igs), are heterodimeric glycoproteins produced by B cells during the adaptive immune response. The diversity of antibody responses necessary to recognize and neutralize a wide range of antigens is achieved by recombination and somatic hypermutation of a set of genes (Aires da Silva et al., 2008; Chi et al., 2020). In mammals, the antibody basic structure consists of two identical heavy chains (H) and two identical light chains (L) in a Y-shaped format. The L chains belong to the kappa (κ) or lambda (λ) subtypes and the H chains to the α, δ, ɛ, γ or μ isotypes. Antibodies are divided into five different classes or isotypes: IgA, IgD, IgE, IgG, and IgM, based on sequence and length of heavy-chain constant domains, each presenting a specific structure and role in immunological processes. Due to its high prevalence in human serum, importance for immune response and excellent specificity, IgG represents the leading isotype used in immunotherapy. In the IgG class, each heavy chain is constituted by three constant domains (CH1, CH2, and CH3) and one variable domain (VH), while the light chain consists of a single constant (CL) and a variable domain (VL; Figure 1A). The antigen-binding fragment (Fab) is composed by the variable domains positioned into the antibody N-terminus and the CL and CH1 regions. A flexible sequence (hinge region) links this fragment to the CH2 and CH3 domains, components of the fragment crystallizable (Fc; Figure 1A). Several inter-domain disulfide bonds along with the highly conserved intra-domain bonds maintain antibody integrity. On one hand, the variable domains promote antibody specificity and affinity toward antigen mainly through three hypervariable loops, collectively known as complementary determining regions (CDRs). The conformation of the VH and VL chain CDRs result in six hypervariable loop structures (H1, H2, H3, L1, L2, and L3) that form the antigen-binding site. These domains also present four relatively conserved β-sheet strands, the framework sequences, which act as scaffolds that support the CDR loops. On the other hand, the Fc region is composed only by constant domains and plays a major role in mediating antibody effector functions through antibody-dependent cytotoxicity (ADCC) and complement-dependent cytotoxicity (CDC). Furthermore, this IgG region is responsible for the prolonged antibody half-life through a recycling mechanism dependent on neonatal Fc receptor binding (FcRn).

In antibody-based therapies, the goal is to eliminate or neutralize the pathogenic organism or the disease target. Within this context, mAbs can achieve their therapeutic effect through various mechanisms. In some applications, antibody binding can directly and effectively block the activity of many pathogens or targets such as virus or receptors expressed on tumor cells. In other settings, effective treatment requires a more general immune response, and antibodies must boost effector functions, such as ADCC and/or CDC responses. In ADCC responses, upon antibody recognition of an antigen, the Fc domain engages Fc receptors (FcγRs) on the surface of effector cells such as macrophages and natural killer cells that mediate phagocytosis or lysis of the target antigen. In CDC responses, antibodies promote directly target cell death through the development of a complement chain membrane attack complex (Aires da Silva et al., 2008). These effector functions contribute to the therapeutic efficacy of several antibodies in clinical settings in which the destruction of target cells is desired, such as the removal of tumor cells or virally infected cells (Reff et al., 1994).

## Antibody fragments and single-domain antibodies as promising therapeutics

The vast majority of mAbs on the market are in the form of a full IgG. These molecules have a long half-life and the ability to induce effector functions if necessary. However, the use of a whole IgG has significant limitations, such as, high molecular weight of these molecules, limiting their penetration into solid tumors, and high production costs. By selecting specific antibody domains, some properties such as half-life, tissue penetration, and affinity can be managed according to the intended application. So, different antibody fragments have been explored in the past years. The use of antibody fragments instead of antibodies in their full structure is associated with numerous advantages. Despite the removal of some domains, these fragments retain the specificity of the original immunoglobulin and for this reason, the antigen recognition remains unchanged. The smaller size of these molecules allows the access to hidden epitopes that would not be available to larger molecules such as full-size mAbs. In addition, due to the lack of glycosylation, antibody fragments can be produced using prokaryotic expression systems at a lower production cost. The lack of the Fc domain is also associated with lower immunogenicity, no antibody-dependent enhancement (ADE) effect stimulation, and lower nonspecific uptake in tissues that highly express Fc receptors (Holliger and Hudson, 2005). Therefore, to avoid Fc-associated effects in some clinical settings and address the size limitations of IgGs, smaller antibody molecules such as the antigen-binding fragment (Fab) or the single-chain variable fragment (scFv) may be produced and become more attractive as therapeutics and diagnostic agents (Figure 1B). Before the development of recombinant DNA technologies and protein engineering methods, Fabs could only be generated through proteolytic cleavage of antibodies. Currently, it is possible to clone the variable (V) and the constant (C) domain genes of the light (L) and heavy (H) chains of an IgG molecule to obtain a recombinant Fab (Figure 1B). Similarly, cloning the VH and VL variable genes of an IgG alone permits the production of an antibody in the scFv format. In this case, the scFv is constituted by a VL-VH pair connected by a flexible peptide linker, in order to improve antibody folding and stability (Figure 1B; Holliger and Hudson, 2005). Currently, several antibodies in the Fab and scFv format have been approved. For instance, Abciximab, Ranibizumab, Certolizumab pegol, and Idarucizumab have been approved in the Fab format, and Blinatumomab and Brolucizumab in the scFv format. With these antibody fragments already on the market and with several under development it is clear that antibody fragments are becoming an increasingly important class of therapeutic agents. Single-domain antibodies (sdAbs) are another class of antibody scaffolds that have gained attention as promising therapeutics.

**Figure 2:**
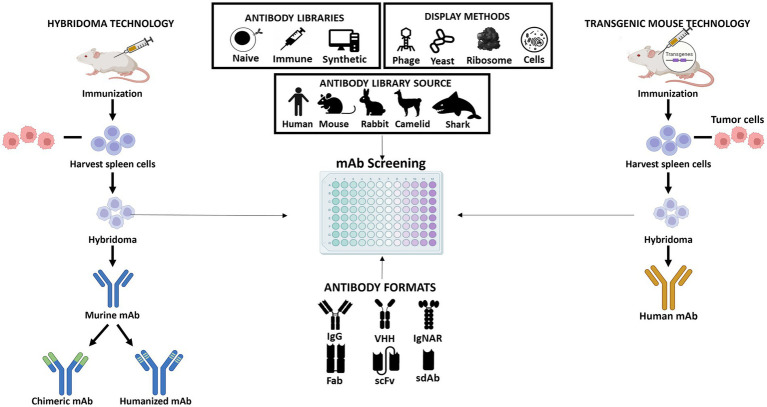
Schematic representation of the different approaches for producing monoclonal antibodies. Hybridoma technology allows the production of murine antibodies which can be modified to generate chimeric or humanized antibodies (shown at left); The procedure of obtaining human antibodies using genetically engineered mice with human immunoglobulin genes (shown at right); The central part of the figure shows the different antibody libraries (naïve, immune, and synthetic) which can be constructed from different sources (human, mouse, rabbit, camelid, or shark). The antibodies that are part of the libraries can be presented in different formats (IgG, VHH, IgNAR, Fab, scFv, or sdAb) and their screening is performed using different display technologies (phage, yeast, ribosome, or cell display).

sdAbs are the smallest functional antigen-binding fragments of an antibody that can be isolated from conventional IgGs or obtained from naturally occurring antibodies devoid of light chains that were discovered in two types of organisms, the camelids (VHH, from camels and llamas) and cartilaginous fish (V_NAR_, from wobbegong and nurse shark; Hamers-Casterman et al., 1993; Greenberg et al., 1995; Muyldermans et al., 2001; Holt et al., 2003; Aires da Silva et al., 2004; Holliger and Hudson, 2005; Figure 1B). Due to their small size, sdAbs in the VH, VL, VHH, or V_NAR_ formats show improved tissue penetration and are able to reach targets that are not easily accessible by conventional antibodies or antibody fragments, such as enzyme active sites, hidden epitopes, or canyons in receptor molecules. Moreover, similar to Fab and scFv, sdAbs lack the Fc domain of a full IgG antibody, presenting a low nonspecific uptake in tissues that highly express Fc receptors. Additional important advantages include their high stability, low immunogenicity, and lower manufacturing costs (Holliger and Hudson, 2005). These unique characteristics and features make sdAbs promising candidates for the development of a new generation of antibody-based therapeutics. Indeed, several sdAbs have been developed and evaluated in different clinical trials and Caplacizumab is a first-in-class that was recently approved for treatment of acquired thrombotic thrombocytopenic purpura (aTTP), a rare disease characterized by excessive blood clotting in small blood vessels (Morrison, 2019). Caplacizumab is a sdAb in the VHH format that works by binding to the A1 domain of von Willebrand factor (VWF) and blocking platelets from binding to VWF and aggregating.

The small size of antibody fragments and single domains improves their ability to penetrate tumors and leads to rapid clearance from circulation through the kidney. In some therapeutic applications, rapid clearance is beneficial. However, in other cases, it is desirable to increase the half-life of the antibody. This can be achieved by linking the antibody fragment or a single domain to polyethylene glycol (PEG; Kontermann, 2011). This process, named PEGylation, increases the serum half-life and simultaneously reduces the immunogenicity of proteins by chemical coupling of PEG to amino groups on the antibody. Although this approach is an industry-established method, several studies also indicate that PEGylation can lead to reduced antigen binding and bioactivity of the PEGylated protein. As an alternative, other half-life extension methods have been developed by exploring the neonatal Fc receptor (FcRn) mediated recycling, responsible for the long half-life of the human immunoglobulin G (IgG, ∼21 days) and human serum albumin protein (HSA, ∼19 days). Essentially, these strategies are based on the fusion to the IgG Fc region, fusion to serum albumin and fusion to anti-albumin antibodies or peptides. Another promising strategy that has been explored is to fuse the antibody with naturally occurring albumin-binding domains (ABD) found in protein G of certain streptococcal strains (Dennis et al., 2002; Stork et al., 2007; Kontermann, 2011; Sleep et al., 2013; Cantante et al., 2017).

## Engineering multivalent, bispecific, and new generation of antibodies

Converting whole IgG antibodies into Fab, scFv, and single-domains might lead to a decrease in the antigen-binding activity due to the loss of avidity. Nonetheless, this loss in binding activity can be compensated by engineering multivalent antibody fragments. Over the past years, several studies have been applied to construct multivalent antibodies through the use of either chemical or genetic cross-links. These engineered antibodies span a molecular-weight range of 60–150 kDa and valences from two to four binding sites. For instance, several Fab fragments have been chemically cross-linked into di-and trivalent multimers, leading to an increased functional affinity (Casey et al., 1996, 2002; Weir et al., 2002). On the other hand, several strategies have been devised to genetically develop multimeric scFvs, by simple engineering the size of the amino acid linker between the VH and VL domains (Huston et al., 1993; Holliger and Hudson, 2005). These antibody engineering methods have led to the formation of dimeric molecules (diabodies, ~60 kDa), trimers (triabodies, ~90 kDa) or tetramers (tetrabodies, ~120 kDa). All these multivalent antibody fragments are usually generated as monospecific molecules. Antibody engineering strategies have also been applied to generate bispecific antibodies (BsAb). These types of antibodies bind to two different antigens, or two different epitopes on the same antigen (Hudson and Souriau, 2003). The high interest in these molecules occurs because many diseases involve parallel signal pathways or multiple inhibition of different receptors or ligands. In fact, the simultaneous blocking of different targets can limit disease progression as well as the development of possible mechanisms of resistance derived from single or combinatory therapy. BsAb that mediate cytotoxicity by recruiting and activating CD3+ T immune cells are also a promising immunotherapeutic strategy for treatment of hematological malignancies and solid cancers. BsAbs can be developed and engineered in the IgG format as well in different antibody fragment constructs. Each of these molecules, depending on their structure, demonstrate different properties of valency for antigens, half-life and in some cases effector functions (Spiess et al., 2015). Currently, several BsAbs are under development for treatment of cancer and other diseases, and five have already received FDA approval: blinatumomab, emicizumab, amivantamab, tebentafusp and faricimab.

Antibody-drug conjugates (ADCs) and chimeric antigen receptor (CAR)-T-cell-based therapeutics, are two other promising classes of engineered antibodies that have emerged as promising therapeutic strategies in oncology. ADCs comprise an antibody conjugated to a highly cytotoxic compound *via* a chemical linker that directed toward a target antigen expressed on the cancer cell surface, reducing systemic exposure and therefore toxicity (Sievers and Senter, 2013). CAR-T-cell-based therapeutics consist of an antibody-derived targeting domain fused to T-cell signaling domains that, when expressed by a T-cell, endows the T-cell with antigen specificity determined by the targeting domain of the CAR (Sterner and Sterner, 2021). Currently, 12 ADCs and 6 CAR T-cell therapies have been approved by the FDA and EMA. With these advances in modern biotechnology and miniaturization and multifunctionalization of antibodies the market for therapeutic antibody drugs has experienced explosive growth. As a result, therapeutic antibodies have become the predominant class of new drugs developed in recent years for the treatment of various human diseases, including many cancers, autoimmune, metabolic, and infectious diseases.

## Selection and screening of monoclonal antibodies

The mouse hybridoma technology was an important step in the development of mAbs. A mouse hybridoma is a hybrid cell produced by injecting a specific antigen into a mouse, collecting the antibody producing cell from the mouse’s spleen, and fusing it with a long-lived cancerous cell (myeloma). The resulting hybrid cell can be isolated and expanded producing many identical offsprings. Each of these daughter clones will secrete over a long period of time, the immune cell product, the antibody. A B-cell hybridoma secretes a single specific antibody known as a monoclonal antibody (Figure 2). Following the discovery of the Nobel-prize winning hybridoma technology, the door was opened for the use of mouse antibodies as human therapeutics. However, as mentioned above, murine mAbs present several properties that limit their clinical utility. Therefore, over the past few years several antibody screening methods have been explored to develop chimeric, humanized, and human high-affinity antibodies. Some of these platforms consist in display technologies, such as phage display, ribosome display, yeast display, bacterial display, mammalian cell surface display, and transgenic mice platforms that express human immunoglobulin genes. The phage display is the most commonly used *in vitro* technology to generate recombinant therapeutic antibodies from different sources and antibody formats (Figure 2). Indeed, over the past years, phage display methodology has been refined and advanced to enable the discovery of antibodies against well-validated targets as well challenging targets and unmet medical needs. Up to date, 14 FDA/EMA-approved therapeutic antibodies have been developed using phage display (Supplementary Table 1), including the world best-selling antibody adalimumab (Alfaleh et al., 2020). In the next section, we will first summarize a general overview of the classic *in vitro* phage display technology and then discuss the state-of-the-art of *in vivo* phage display methodologies as a promising innovative strategy for the improvement of antibody targeting and drug delivery properties.

## Phage display technology

**Figure 3:**
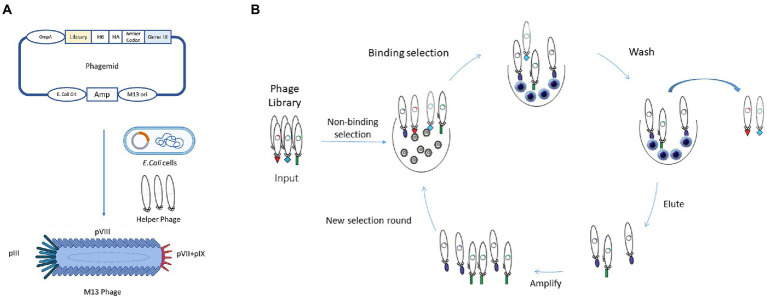
Schematic representation of *in vitro* Phage Display Technology. **(A)** In phage display systems, antibody genes are linked to the amino terminus region of the phage minor coat protein pIII, as shown in the phagemid. When expressed, mature phages will incorporate the encoded fusion product, creating a link between antibody genotype and phenotype. **(B)** *In vitro* phage display, selection is composed of several steps: coating of the antigen or preparation of the cells; incubation of phage repertoire with antigen; washing to remove non-specific phages; and elution and reamplification of antigen-specific phages. The stringency of selection could be increased by the increase in the number of washing steps.

Phage display was firstly described in 1985 when George P. Smith demonstrated that a filamentous bacteriophage f1 was capable of displaying a fusion protein on the virion surface after the insertion of a foreign DNA fragment into the phage coat protein gene (Smith, 1985). In the early 90s, this technology was further developed and improved for the display of antibodies, mainly by the groups of Winter and McCafferty at the Laboratory of Molecular Biology (Cambridge, United Kingdom), and by the groups of Lerner and Barbas at The Scripps Research Institute (La Jolla, United States; McCafferty et al., 1990; Barbas et al., 1991). In these systems, the antibody genes are linked to the amino-terminus region of the phage minor coat protein pIII. Thus, when expressed, during normal phage biogenesis, mature phage particles incorporate the encoded fusion product, linking the antibody genotype and phenotype (Figure 3). By producing phages that express on their surface antibodies while possessing in their genome the antibody encoding gene, phage display allows the enrichment of antigen-specific phage antibodies, using immobilized or labeled antigens (Winter et al., 1994; Hoogenboom, 2002). Four main steps are part of the process of a classic phage display selection: coating of antigen; incubation of phage repertoire with antigen; washing to remove non-specific phages; and elution and reamplification of antigen-specific phages (Hoogenboom, 1997, 2005; Griffiths and Duncan, 1998; Figure 3). After the incubation of the phage library with the antigen, the unbound phages are washed away. This step is crucial to avoid the selection of non-specific binders. To obtain high-affinity phages, the stringency can be incremented in each round by increasing the number of washing steps. Moreover, the increase in stringency can also be attained through the modification of the washing buffer, for example by adding detergents (Smith and Petrenko, 1997). Regarding the elution step, different conditions can be used, such as changing the pH level, proteolytic cleavage or competition with free antigens. To facilitate cleavage, some libraries possess a cleavage site, such as trypsin, between the antibody and the pIII protein (Ward et al., 1996; Kristensen and Winter, 1998). In general, 3 to 6 rounds of binding, elution, and amplification are sufficient to recover antibodies with high specificity and affinity (Hoogenboom, 1997, 2005; Griffiths and Duncan, 1998).

The same panning principle and protocol can be applied to run a phage display to select antibodies against antigens expressed on whole cells, liposomes, virus-like particles or other systems (Kirsch et al., 2008; Lipes et al., 2008; Dominik and Kossiakoff, 2015; Jones et al., 2016; Stark et al., 2017).

Various types of phages can be used as phage display systems and depending on the type of phage, the systems can be classified as filamentous M13, T7, bacteriophage lambda (λ phage) or T4 phage display systems. Filamentous phages, namely M13, are the most common used in phage display, as they do not lyse cells during their lifecycle. As shown in Figure 3, M13 consists of a circular ssDNA and five coat proteins (pVIII, pIX, pVII, pVI, pIII). There are two major types of M13 libraries based on the peptide or antibody fusing positions of phage, pIII and pVIII library. Both contain an N-terminal signal sequence that directs them to the inner bacterial membrane before phage assembly. The size of the pIII coat protein is about 406 amino acids long and is involved in phage-host interaction during infection. The great advantage of the pIII phage library is that it can display large foreign proteins on its surface with one to five copies, that makes the pIII system the most commonly used in phage display libraries. The main advantage of this phagemid system is their smaller size and ease of cloning (Bábíčková et al., 2013; Tan et al., 2016). With the growing use of antibody fragments and single-domain antibodies, grows the necessity to improve the phage display systems. For instance, in order to improve the number of scFvs presented on filamentous phage particles, a new kind of phages were developed, known as hyperphages. Hyperphages have a pIII phenotype and are able to infect *E. coli*, however, lack the functional pIII gene, which means that the phagemid-encoded pIII-antibody fusion is the source of pIII in phage assembly. These unique features increase the phage particles that are carrying the scFv on their surface, increasing the antigen-binding activity more than 50% compared to 3% when conventional phages were used (Rondot et al., 2001; Pande et al., 2010).

A critical feature to maximize the chance of discovering an antibody with the desired characteristics is the type of antibody source used. There are three main types of antibody libraries: immune, naïve, and synthetic (Winter et al., 1994; Hoogenboom, 2002; Kügler et al., 2018). Immunized libraries use as antibody source the lymphoid tissues of a donor that was previously immunized with a specific antigen or, in some instances, individuals with a particular disease, such as cancer or a particular infection. Normally, this type of library results in higher-affinity antibodies than those generated from hybridomas and naïve libraries, as gene fragments go through an *in vivo* maturation process (Winter et al., 1994; Griffiths and Duncan, 1998; Hoogenboom, 2002; Bradbury and Marks, 2004). However, as immunizations are required, immune libraries include certain limitations. These disadvantages are related with the time required to immunize animals, unpredictability of the immune response to the target antigen, lack of immune response to some antigens and a new library must be constructed for each antigen. Moreover, due to ethical concerns, human libraries can only be generated from patients B cells. On the other hand, issues related with immunizations are not present in the naïve libraries. These types of libraries are recovered from large naïve repertoires of antibody fragments from non-immunized donors and represent the germline diversity of the antibody repertoire. Due to their characteristics, naïve libraries have some advantages over immunized libraries. Foremost, antigen-specific antibodies can be produced without the need of previous immunizations, being able to get antibodies against self, non-immunogenic or toxic antigens. Furthermore, unlike immune libraries, naïve libraries can be used for all antigens, if large enough. At last, antibody generation is a much quicker process and in less than 2 weeks human antibodies can be isolated (Winter et al., 1994; Hoogenboom, 2002). The main disadvantage is the large size of the naïve library. The large size makes the exact nature of V-gene repertoire largely unknown and uncontrollable (Maynard and Georgiou, 2000). Additionally, one of the main problems reported are the poor expression and toxicity to the host bacteria with antibodies isolated from naïve libraries. This is due to the fact that antibody genes are representative of the human immunological repertoire and because of that there is no guarantee that the clone obtained can be expressed in bacteria (Maynard and Georgiou, 2000). The problems reported may be bypassed by using synthetic antibody libraries. Synthetic libraries are *in vitro* created using oligonucleotides that introduce areas of complete or tailored degeneracy into the CDRs of one or more V genes. The degeneracy introduced into specific codon positions of synthetic oligonucleotides allows the control of the degree of randomization. The first synthetic library was constructed by Marks et al. (1991). For this purpose, a repertoire of human VH genes from 49 human germline VH-gene segments *in vitro* rearranged was developed to create a synthetic CDR3 of five to eight residues. For phage display, rearranged VH genes were cloned with a human Vλ3 light chain as single-chain variable fragments (scFvs). The selection resulted in the isolation of many antibodies against haptens and one against a protein antigen. These great results were part of the first proof of concept for synthetic antibodies. Thereafter, to cover the natural length diversity of the loop of the CDR3, the diversity was expanded from 4 to 12 residues (Nissim et al., 1994). The success of the expanded library showed the importance of diversity in a longer CDR-H3 loop. To improve the synthetically rearranged VH gene repertoire of Nissim et al. (1994) and Griffiths et al. (1994) developed a novel system to add light-chain diversity to the previous VH gene repertoire. In this study, 26 human germline Vk and 21 germline Vλ segments were assembled into complete V genes using PCR with CDR3 loops partially randomized to mimic the diversity generated by V-J gene recombination *in vivo.* In order to create a large synthetic repertoire of Fab fragments displayed on a filamentous phage, the heavy and light-chain V-gene repertoires were combined on a phage vector in bacteria using the lox-Cre-site-specific-recombination system. This resulted in the development of antibodies with affinities similar to those obtained from a secondary immune response in mice (Griffiths et al., 1994). A summary of some of the phage display antibody libraries used in the discovery of therapeutic antibodies is shown in Table 1.

**Table 1:** Phage display antibody libraries used in the discovery of therapeutic antibodies.

Library name	Company/laboratory	Repertoire	Display format	Size	References
-	Dyax	Naïve	Fab	3.7 × 10^10^	de Haard et al., 1999
BMV	CAT	Naïve	scFv	1.4 × 10^10^	Vaughan et al., 1996
CS	CAT	Naïve	scFv	1.29 × 10^11^	Lloyd et al., 2009
BMV	CAT	Naïve	scFv	1.2 × 10^11^	Groves et al., 2006
HuCAL	Morphosys’s	Synthetic	Fab	2.1 × 10^9^	Knappik et al., 2000
HuCAL GOLD	Morphosys’s	Synthetic	Fab	1.6 × 10^10^	Rothe et al., 2008
HuCAL PLATINUM	Morphosys’s	Synthetic	Fab	4.5 × 10^10^	Prassler et al., 2011
pIX V3.0	Janssen Bio	Synthetic	Fab	3.0 × 10^10^	Shi et al., 2010
XFab1	Xoma	Naïve	Fab	3.1 × 10^11^	Schwimmer et al., 2013
XscFv2	Xoma	Naïve	scFv	3.6 × 10^11^	Schwimmer et al., 2013
PHILODiamond	ETH Zurich	Synthetic	scFv	4.1 × 10^10^	Weber et al., 2014
ALTHEA Gold Libraries	GlobalBio/ADL	Semi-synthetic	scFv	2.1 × 10^10^	Valadon et al., 2019
HAL9/10	Technische Universität Braunschweig	Naïve	scFv	1.5 × 10^10^	Kügler et al., 2015

Library size corresponds to the number of colony forming units (cfu) after the transformation of E. coli with the corresponding DNA library.

## *In vivo* phage display

**Figure 4:**
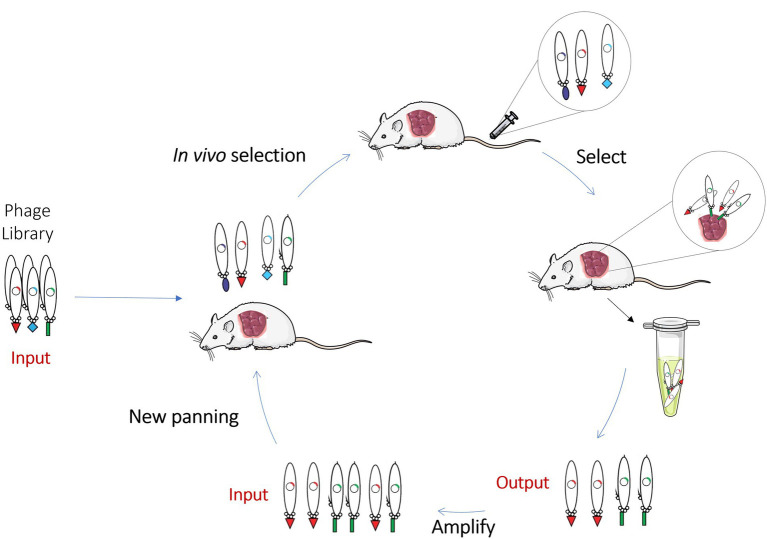
Schematic representation of *in vivo* Phage Display. *In vivo* phage display selects phage libraries using a living animal. In this methodology, the library is directly injected into animals and antibodies are allowed to bind directly to the specific organ or tissues. Non-binding phages are washed and, in the end, animals are euthanized, and the desired organs collected to recover the phages. This scheme contains a representation of an *in vivo* experiment performed on a xenograft mouse model where the phages are recovered from the tumor.

With the emergence of new classes of therapeutic antibodies and novel disease targets, it has become increasingly necessary to improve the conventional phage display methodology to generate better molecules against challenging targets. Thus, to fulfill these unmet needs and to select best-in-class antibodies against antigens in their native conformation and *in vivo* settings, innovative ways to perform the phage display selection have been developed, namely the *in vivo* phage display. *In vivo* phage display consists in the selection of phage libraries using biopannings in living animals. This approach is identical to the *in vitro* phage display, however, the main difference is that the phage library is directly intravenously injected into animals and the phages are allowed to circulate in order to allow the antibodies expressed at the phage surface to bind directly to the specific target, organ or tissues. In the end, animals are perfused to wash the unbound/unspecific phages, euthanized and the desired organs are collected to recover the phages (Figure 4). With this methodology antibodies are selected in the complicated milieu of the animal based on desired pharmacokinetic and targeting specificity properties. In order to have a well-designed *in vivo* phage display experiment, it is necessary to be aware of some parameters that can modulate the expected results. Some of these are related to phage survival and pharmacokinetics in the living animal and the route of administration that can limit the success *of in vivo* selection. These key aspects of an *in vivo* phage display panning will be overviewed and discussed below.

### Experiment design of *in vivo* phage display

#### Phage library and phage survival

The survival of phage particles in the animal is a critical issue when an *in vivo* phage display is performed. It is well known that the immune system and, in particular, the reticuloendothelial system (RES) have a preponderant role in the phage uptake. In fact, this is clearly demonstrated by the high uptake observed in the liver and spleen. To better understand these limitations, various studies compared the survival time of phages in different mice models. Zoe et al. described that 5 min after injection, the percentages of phages in circulation decreased in all mice strains and the highest level of phage particles was detected at 5 min post-injection in CF-1 and SCID mice, and 15 min post-injection in nude mice (Zou et al., 2004). Furthermore, a minor phage accumulation in the spleen was found in the immune-compromised nude and SCID strains, suggesting a facilitated leakage and reducing of non-specific trapping by RES (Zou et al., 2004). Interestingly, Srivastava and colleagues screened a phage library in 6 different mice models and suggested that the main cause for phage degradation was related to B-cell interaction (Srivastava et al., 2004). However, Zou *et al.* described contradictory results, reporting higher phage survival in the athymic mice strain that possess B-cells (Zou et al., 2004). Additionally, Srivastava et al. evaluated phage recovery after 5 min post-injection and verified that no more than 50% was recovered, while confirming that this value decreased with incubation time (Srivastava et al., 2004). Overall, these *in vivo* phage display findings supported incubation times ranging between 5 to 15 min after intravenous (i.v.) administration. Altogether, these studies concluded that the inclusion of a phage library circulation time optimization study is recommended for each type of phage and animal model before initiating an *in vivo* phage display experiment (Molenaar et al., 2002).

#### Route of administration

The route of administration is another important issue to be considered in the design of an *in vivo* phage display experiment. Different routes have been used for phage library administration, and each route may influence the uptake to the target tissue or organ. Due to the rapid systemic exposure of the phage particles, intravenous injection is the route most widely used. However, before choosing the preferential route of administration, it is advisable to consider the targeted organ or tissue. This is particularly true in organs such as the brain that have a less effective systemic delivery due to the blood–brain barrier. The complexity of the target is another problem to have in consideration. For example, in the lungs the vasculature is heterogeneous which can hinder the identification of lung specific receptors by systemic administration of the library. To overcome this problem, phage administration *via* intratracheal instillation may be executed (Wu et al., 2003). Another route with great potential is intestinal and oral administration, considering the important role that the gastrointestinal tract may play in drug diffusion and metabolism. Nevertheless, few studies have reported the use of this route, probably due to the expected degradation caused by digestive juices. Even so, Duerr et al. performed an experiment to recover peptides capable of crossing the intestinal mucosal barrier. Based on the assumption that spleen accumulation is due to the translocation of the trans-mucosal barrier, phages were retrieved from the spleen. However, no consensus sequence in the phages recovered was discovered (Duerr et al., 2004). A few years later, another study from Hamzeh-Mivehroud et al. (2008) obtained similar results, proving that trans-mucosal transport is non-specific (Hamzeh-Mivehroud et al., 2008). Another study of Akita et al. (2006) aimed to identify new peptides for peritoneal tumors of gastric cancer. For this purpose, an *in vivo* intraperitoneal biopanning was performed. The phages were injected directly into the abdominal cavity of mice presenting peritoneal tumors of human gastric cancer. This work allowed to identify peptides for the treatment of peritoneal metastasis of gastric cancer (Akita et al., 2006). Overall, these findings show that the selection of an administration route near the target tissue can have some advantages, such as the reduced uptake by other tissues (Bábíčková et al., 2013).

### *In vivo* phage display discovery applications

#### *In vivo* phage display of peptides

*In vivo* phage display was first described by Pasqualini and Ruoslahti (1996). In this study, a peptide that targets the brain and kidneys was discovered using phage peptide libraries. This first study obtained promising results, culminating in the identification of the tripeptide SRL that specifically targets the brain and the CLPAVASC peptide amino acid sequence that targets the kidney (Pasqualini and Ruoslahti, 1996). Years later, another group used the same methodology to isolate other tripeptide motifs to the brain and kidney tissues (Kolonin et al., 2006). Since then, *in vivo* use of phage display has allowed the identification of several receptor-ligand pairs in malignant and benign disease contexts and different animal models. Interestingly, in 2002 the first *in vivo* phage display in humans was performed by Arap et al. (2002b). In this work an *in vivo* screening of a phage displayed peptide library was performed to investigate the molecular diversity of receptors specific of human vascular beds. For that, a peptide phage library was intravenously infused, and 15 min later tissue biopsies were recovered to retrieve the phages from various organs. A total of 47,160 peptide motifs were then shown to be localized to different organs. This large-scale screening indicated that the tissue distribution of circulating peptides was nonrandom. To validate the specificity of the isolated peptides, the interaction of an IL-11 peptide mimic isolated from the phage display screening to interleukin-11 receptor (IL-11R) of normal prostate endothelium and epithelium tissue was confirmed (Arap et al., 2002b). Later, IL-11R was validated by Zurita et al. (2004) as a molecular target for prostate cancer therapeutics. Afterward, the library obtained by Arap et al. (2002b) was further screened in two subsequent cancer patients to uncover ligand-receptors common or specific to certain vascular beds. These results allowed the identification of four native ligand-receptors, three of which have not been previously reported. Two are expressed in several tissues (integrin α4/annexin A4 and cathepsinB/Apolipoprotein E3) and the other two have a specific distribution in normal tissues (prohibitin/annexin A2 in white adipose tissue) or cancer (RAGE/leukocyte proteinase-3 in bone metastases; Staquicini et al., 2011). Altogether, these studies validated a functional screening strategy based on an *in vivo* phage display for vascular ligand-receptor mapping, aiming to identify potential markers for biotechnology and medical applications.

Another study conducted by Arap et al. (2002a) identified a new peptide through an *in vivo* screening of phage-displayed random peptide libraries targeting the prostate vasculature. One of the selected peptides homed to prostate tissue 10 to 15 times more than other organs, and was therefore coupled to a proapoptotic peptide for the development of a drug delivery system. This proapoptotic peptide is responsible for the disruption of mitochondrial membranes and has shown antitumoral efficacy in prostate cancer prone transgenic mice. Similarly, treatment of prostate proned transgenic mice with this drug delivery system demonstrated that the chimeric peptide inhibited cancer development in this animal model (Arap et al., 2002a). Similar methodologies have been applied in the identification of peptides targeting other solid cancers, such as breast cancer. For example, an *in vivo* biopanning with a phage-displayed peptide library allowed the recognition of a cyclic nonapeptide that selectively homes normal breast tissue and identified aminopeptidase P as the respective receptor. Relevantly, this study also confirmed the binding of this novel peptide to the vasculature of hyperplastic and malignant lesions in transgenic breast cancer mice, suggesting that this peptide could be used to selectively target breast tissue and precancerous breast lesions (Essler and Ruoslahti, 2002). In turn, Laakkonen et al. (2004) aimed to investigate tumor angiogenesis related markers. For that purpose, this work designed an *ex vivo* and *in vivo* phage display to select peptides that favor tumor-homing targets accessible to circulating phages with the exception of blood vessels. Screening was performed on MDA-MB-435 breast carcinoma xenografts and enabled the recovery of multiple copies of a cyclic-9-amino-acid peptide, LyP-1. Gathered data revealed that LyP-1 also recognized tumor cells derived from an osteosarcoma xenograft mouse model, and spontaneous prostate and breast cancer in transgenic mice. LyP-1 peptide labeled with fluorescein was detected in tumor structures positive for three lymphatic endothelial markers and negative for three blood vessel markers, suggesting that this peptide may target tumor lymphatic vessels. Moreover, systemic treatment of breast tumor xenograft mice with this LyP-1 peptide was performed. Curiously, tumor treated samples showed foci of apoptotic cells in the absence of lymphatic vessels, revealing a surprising antitumoral activity of this LyP-1 peptide. Another study implemented an *in vivo* screening of a phage-displayed peptide library to discover peptide ligands that target human ovarian cancer, making it a promising candidates for drug delivery (Ma et al., 2013). This work allowed to isolate a novel peptide WSGPGVWGASVK targeting ovarian cancer derived tumor cells and angiogenic endothelial cells. Altogether these studies clearly demonstrate that *in vivo* phage display selection is a powerful platform for the discovery of cancer targeting peptides, with suitable stability and biodistribution. However, most of these peptides target the tumor vasculature rather tumor cells or tissues. Thus, there is a pressing need to improve phage display selection to identify peptides with the ability to extravasate vessels, penetrate tumors and bind target cells while presenting adequate pharmacokinetic properties. To tackle these issues, Soendergaard et al. (2014) devised a two-tier method that includes *in vivo* selection rounds in xenografted SKOV-3 tumor-bearing nude mice followed by *in vitro* screening on cultured tumor SKOV-3 cells. These studies, resulted in the selection of a specific peptide—RSLWSDFYASASRGP (J18)—with good tumor uptake.

The use of a parallel *in vivo* and *in vitro* phage display selections was also employed in the identification of novel tumor-homing activatable cell-penetrating peptides. For the *in vivo* panning, phages displaying a library of unique activatable cell-penetrating peptides were injected into tumor-bearing mice and cleaved phage were isolated from homogenized tumors. Simultaneously, an *in vitro* selection consisting in the sequential exposure of phage to normal and tumor tissue sample extracts and its subsequent isolation from uncleaved and cleaved phages was carried out. Selected sequences were synthesized as fluorescently labeled peptides, and tumor-specific cleavage was confirmed by digestion with tissue extracts. The most promising cleaved peptide presenting the sequence RLQLKL showed to accumulate within primary and metastatic tumors in a protease-dependent manner. This candidate was therefore selected to detect protease activity in tumors, directing therapeutic or imaging agents (Whitney et al., 2010). Finally, Veleva et al. (2011) group designed a strategy to identify ligands from a phage display random peptide library, selective for cells homing angiogenic tumors derived from circulating bone marrow, that included an *in vitro* phage display selection on blood outgrowth endothelial cells followed by an *in vivo* selection based on homing of bone marrow-bound phage to angiogenic tumors. This dual-functional strategy enabled the isolation of the peptide containing the QFPPKLTNNSML sequence. Further experiments confirmed the ability of this peptide to deliver *in vivo* payloads to angiogenesis sites, reinforcing its clinical potential. Overall these studies described herein paved the way for *in vivo* phage display selections and are summarized in Table 2.

**Table 2:** Summary of peptides discovered by *in vivo* phage display.

Sequence	Animal model	Target	Tissue/organ	References
SRL	Balb/c mice	Unknown	Brain	Pasqualini and Ruoslahti (1996)
CLPAVASC	Balb/c mice	Unknown	Kidney	Pasqualini and Ruoslahti (1996)
LGG	C57BL/6 mice	Unknown	Brain	Kolonin et al. (2006)
SMSIARL	CD1 mice	Unknown	Prostate vasculature	Arap et al. (2002b)
CPGPEGAGC	CD1 mice	Aminopeptidase P	Breast cancer	Essler and Ruoslahti (2002)
LyP-1	Balb/c mice	Unknown	Breast cancer	Laakkonen et al. (2004)
WSGPGVWGASVK	Balb/c mice	EGFR	Ovarian Cancer	Ma et al. (2013)
RSLWSDFYASASRGP	Nude mice	Unknown	Ovarian cancer	Soendergaard et al. (2014)
RLQLKL	PytM mice	Unknown	Cancer	Whitney et al. (2010)
QFPPKLTNNSML	C57BL/6 mice	Unknown	Lewis lung carcinoma	Veleva et al. (2011)

#### *In vivo* phage display of antibodies

Although *in vivo* phage display was initially explored for identifying new peptide sequences that revealed interactions with natural ligands and for the recognition of known or new targets, mAbs have also gained considerable attention due to their many advantages over other binders, namely associated with their high specificity and affinity for antigens and therapeutic properties. Within the antibody fragment repertoire, scFvs and sdAbs have been the antibody formats mainly selected and explored for *in vivo* phage display screenings. Within this context, Johns et al. (2000) described for the first time the development of an *in vivo* phage-scFv display selection procedure in the mouse. This study demonstrated the potential for the selection of scFvs against the murine thymus from a phage-scFv library, opening the possibility of implementing the same method to other models. Briefly, a scFv phage library was injected into a thymic murine model and then phages were recovered from the thymus after four rounds of selection. The specificity of some clones was tested and, in the end, CDR3 sequences of two clones were identified. The authors at the time were unable to identify the targets; however, it was reported that one scFv antigen was solely expressed on the thymic endothelium, while the second was present on both thymic endothelium and the perivascular epithelium. Later, Robert et al. (2006) combined an *in vivo* biopanning of a scFv antibody library with a high-throughput screening to identify human antibodies that target lesions developed early in atherosclerosis. First, the *in vivo* selection was performed in Bourgogne brown rabbits submitted to an atherogenic diet with 0.3% of cholesterol. Then, specificity of the selected scFv was confirmed in homozygous ApoE gene-inactivated mice. To prevent the elimination of scFvs against rare epitopes, only one round of selection was performed. This work allowed to select two scFv clones with high affinity to the early atherosclerotic lesions. Years later, another study of the same group performed an *in vivo* phage display using a human phage scFv antibody library injected into a high-fat-diet-fed ApoE^−/−^ mice. Three different fractions were recovered from the aortic tissue: endothelial cell surface binding phages, and extravasated and internalized phages. Three *in vivo* pannings were conducted and six clones were selected. The results obtained allowed to conclude that *in vivo* phage display in hypercholesterolemic animals enables the identification of scFvs specific to atherosclerotic endothelial and subendothelial tissues, and biomarkers associated with lesions (Deramchia et al., 2012b). Following this study, another study was conducted to evaluate molecular components involved in atherosclerosis. For that, scFv antibody fragments from a semi-synthetic human antibody library were injected in a rabbit model of atherosclerosis induced by lesions. Following, phages were recovered from the injured endothelium, the underlying lesional tissue and the cells within the intima. Clones were selected based on the presence of key amino acids. With this method, seven scFv antibodies that demonstrated specific binding to endothelial cells and inflamed intima-related regions of rabbit tissue sections were selected. This study proved that sequence-based selection can be a suitable method to select scFv libraries by *in vivo* phage display (Deramchia et al., 2012a). More recently, Hemadou et al. (2018) used an *in vivo* phage display coupled with an high-throughput screening using flow cytometry to screen a human combinatorial library of scFv phages on an atherosclerosis rabbit model. Three *in vivo* pannings were performed and then high-throughput flow cytometry analysis allowed to recover 209 clones specific for atherosclerotic proteins. Immunohistochemistry experiments confirmed the *ex vivo* reactivity of 60% of these scFv-phages to rabbit atheromas. This multitude of atherosclerosis specific scFvs identified opened new perspectives in the discovery of new biomarkers. Recently, the same group reported the selection within the previously retrieved clones, of a promising scFv against a targetable biomarker of atheroma plaque progression in the pathological microenvironment. With that in mind, clones were reformulated as scFv fused to the scFv-Fc and their reactivity against in aorta sections from both animal models of atherosclerosis and human specimens of atherosclerotic arteries was tested using flow cytometry and immunoassays. The identification of the promising P3 scFv antibody antigen allowed to propose galectin-3 as an ideal molecular biomarker that can be targeted for diagnostic purposes. Curiously, galectin-3 has been recently proposed as a high-value biomarker for the assessment of coronary and carotid atherosclerosis (Hemadou et al., 2021). Notably, *in vivo* phage display has also been used in diabetes research field. In this context, two different methods were reported for the selection of antibodies targeting pancreatic islets. A scFv phage library was screened using two distinct approaches – an *in vivo* and *in vitro* screening. In the case of the *in vivo* selection, the scFv phage library was intravenously injected into rats and phages were isolated from the pancreatic islets. In the *in vitro* selection, the scFv library was panned on pancreatic islets directly isolated from rats. This work reported the successful isolation of four scFv antibodies that are selectively internalized into rodent β-cells *in vivo* and bind to human β-cells *in situ*. Furthermore, two additional scFv antibodies targeting islet α-cells were identified. These scFv antibodies were highly specific for their target cells over other tissues. Moreover, these radiolabeled scFv antibodies showed to reliably predict β-cell mass in rodent models of diabetes after *in vivo* administration, thereby representing promising candidates for *in vivo* imaging and determination of β-cell mass in humans (Ueberberg et al., 2009; Ueberberg and Schneider, 2010). As expected, also in cancer, *in vivo* phage display has been explored as a promising discovery platform for antibody libraries. Krag et al. (2006) reported a phase I clinical study where infusion of phage-display libraries in cancer patients were performed to identify tumor-targeting ligands. In this study, eight patients with advanced cancer, including breast, melanoma, and pancreas, were intravenously infused with a phage-displayed scFv library or a peptide library. Following 30 min post-injection, tumors were excised and tumor-targeting phages were recovered. In three patients, serial pannings were performed by infusing phage recovered and amplified from the same patient’s tumor. Importantly, there was no significant toxicity observed in this preliminary study. Furthermore, the patients did not exhibit detectable antiphage serum IgG before infusion and high antibody response was not observed over the 10-day study infusion limit. Overall, the data obtained allowed the identification of several amino acid motifs among tumor-homing phage clones, suggesting selective tumor accumulation. Six different clones were isolated from the scFv library while only one was isolated from the peptide library. Thus, this study confirmed the feasibility of this technology in personalized medicine, making it possible to select patient tumor-binding ligands in a customized manner. Another study identified a new tumor-specific vascular targeting antibody *via in vivo* phage display of a llama VHH sdAb phage library in an orthotopic mouse model of diffuse glioma. C-C7, the VHH identified, recognized a subpopulation of tumor blood vessels in glioma xenografts and clinical glioma samples. The use of C-C7 VHH as bait in yeast-2-hybrid screens made it possible to recognize dynactin-1-p150Glued as its receptor, a novel targetable protein in activated endothelial cells and macrophages. Thus, validating the combinatorial approach of *in vivo* phage display and yeast-2-hybrid screenings as a promising strategy to identify tumor-targeting antibodies and their binding counterparts (van Lith et al., 2016). *In vivo* phage display screenings have also been explored to select antibodies that can cross the blood–brain barrier (BBB) and specifically target the brain. For instance, a naïve human library was used to identify brain targeting scFv antibodies using an *in vivo* phage display selection in a rat model. Two scFvs, namely scFV40 and scFV4, were selected, and their binding and BBB crossing properties were confirmed (Stutz et al., 2018). Our group has also been working toward the discovery of specific antibodies that target the BBB. We have recently immunized rabbits with brain mice endothelial cells and constructed an sdAb immune library that was then used for an *in vivo* phage display selection in a mouse model. After three rounds of *in vivo* phage display, five potential sdAbs were identified with potent brain targeting and BBB crossing properties. Moreover, a superior brain accumulation was observed for the RG3 clone, (0.82 ± 0.05% I.A./g) and, as far as we are aware, positioning it as one of the most competent sdAb in BBB translocation described so far. To demonstrate the brain targeting properties and efficacy of our platform, the RG3 clone was conjugated to liposomes containing a well-known cytotoxic drug. Notably, the RG3-sdAb conjugate liposomes demonstrated an efficient BBB translocation and a potent antitumoral activity against glioblastoma (Aguiar et al., 2021). Our group has also been exploring the potential of *in vivo* phage display to select highly specific and potent ADC molecules. In this case, an immune sdAb library against non-hodgkin lymphoma (NHL) receptors was first submitted to three rounds of an *in vitro* whole-cell phage display. Then, the selected pool was subjected to a final *in vivo* panning on a xenograft mouse model of NHL. The recovered phage pool was submitted to next-generation sequencing and a panel of highly specific candidates were identified. One of the most promising sdAbs was selected and used to be conjugated with a cytotoxic compound to develop a new ADC (Silva et al., 2021). Taking into account the results obtained so far using *in vivo* antibody phage display, it is clear that *in vivo* screenings can be a promising selection strategy for the improvement of antibody targeting and drug delivery for several clinical applications. Table 3 summarizes the *in vivo* phage display described herein.

**Table 3:** Summary of antibodies fragments discovered by *in vivo* phage display.

Name	Format	Animal model	Target	Tissue/organ	References
E3A1	scFv	CBA mice	Unknown	Thymus	Johns et al. (2000)
K3.1	scFv	Bourgogne brown rabbits	Carbonic anhydrase II	Atherosclerosis	Deramchia et al. (2012a); Deramchia et al. (2012b)
P3	scFv	Rabbit	Galectin-3	Atherosclerosis	Hemadou et al. (2018); Hemadou et al. (2021)
SCA A1 and SCA B1	scFv	Rats	Unknown	Pancreatic islets	Ueberberg et al. (2009); Ueberberg and Schneider (2010)
269	scFv	Cancer patients	Unknown	Tumor-targeting ligands	Krag et al. (2006)
C-C7	Llama nanobody	Mice	Dynactin-1-p150Glued	Glioma	van Lith et al. (2016)
scFv 4 and scFv 40	scFV	Rat	Unknown	BBB	Stutz et al. (2018)
RG3	sdAb	CD1 mice	Unknown	BBB	Aguiar et al. (2021)
C5	sdAb	Mice	Unknown	cNHL	Silva et al. (2021)

## Discussion

Since the discovery of hybridoma (Köhler and Milstein, 1975), new perspectives have been launched in the field of antibody research and clinical development. Although promising results have been achieved, as advances in molecular tools and the disadvantages of hybridoma were more evident, new alternatives for antibody development were pursued. Due to its characteristics, display selection technologies have emerged as a promising alternative to traditional methods. Nowadays, phage display is the most widespread and powerful display technology to select antibodies and peptides. Barderas and Benito-Peña (2019) awarded with the Nobel Prize for Chemistry for the development of phage display for peptides and antibodies. The phage display created a fast route for the approval and development of molecules selected by this technique. The biggest advantage of this display method is the linkage between phenotype and encapsulated genotype (Tan et al., 2016). Furthermore, the use of phage display facilitates the cloning process due to the phage’s genome small size and the high efficiency of phage infection. There are still other advantages that can be associated with this methodology, such as the small size of the phages, easy manipulation, safety, low cost of phage preparation and propagation, and the possibility of high-throughput screening of peptides and antibodies (Bábíčková et al., 2013). Additionally, upon the construction of the phage library, phages can been screened against any target antigen within several weeks (Greenwood et al., 1991). However, as with all techniques there are some pitfalls. The capacity of the phage display library and its molecular diversity is limited since it depends on bacterial transformation, phage packing and transmembrane secretion processes (Shim, 2017). Furthermore, the expression system is also limited because not all the sequences can be efficiently expressed in phages, considering that certain proteins require the correct folding to acquire its functions (Alfaleh et al., 2020). Nevertheless, phage display continues to be considered the best tool to detect the protein spatial structure, exploring binding sites between receptors and ligands, as well as the search for ligands with high affinity and biological activity. Despite the great results, out of all the therapeutic molecules approved by the FDA since 2000, only 14 monoclonal antibodies and around 30 peptides were selected using phage display, while hybridoma remains the most used methodology (Alfaleh et al., 2020; Wang et al., 2022). Within this context, it is evident that there is still a need for improvement the selected molecules by phage display so that they can be used in clinical practice. Along with this, and to surpass some of the pitfalls associated with phage display in general, the methodology has been going through some improvements in order to better meet the needs. One of the unmet needs is the increasing necessity for more specific molecules. This specificity can be achieved through *in vivo* phage display. Despite its early development, *in vivo* phage display has gained a prominent place over *in vitro* techniques. *In vivo* phage display allows the screening of molecules on a living animal or human patients, enabling the molecules to be selected in their natural environment which leads to a more specific molecule selection. Indeed, as selection is performed on an *in vivo* milieu, negative selection is naturally performed, excluding the non-specific phages that bind to non-target organs or tissues. Nevertheless, some drawbacks can be attributed to *in vivo* phage display, such as biodegradability of the phages by the immune system, interspecies differences and low reproducibility (Bábíčková et al., 2013). However, as shown in the reviewed studies, these issues can be easily surpassed *via* the optimization of some parameters, such as the time of incubation and the type of models used for panning.

It is important to take in consideration that according to the European Union (EU) Directive 2010/63/EU and the EU Reference Laboratory for Alternatives to Animal Testing (EURL ECVAM) recommendations, the EU and its members are encouraged to promote the generation of new molecules using non-animal methodologies (Gray et al., 2020a, b; Bradbury et al., 2021). These concerns are entirely valid and that is why the *in vivo* phage display can be very useful to reduce animal usage upfront in the subsequent efficacy and safety pre-clinical studies. The *in vivo* phage display has the advantage of selecting the best antibody or protein candidates given its characteristics of specificity, pharmacokinetics and stability. Indeed, with an *in vivo* phage display screening we can identify the best lead candidates *in vivo* and reduce the number of candidates to be tested in pre-clinical studies. Furthermore, a single round of *in vivo* phage display can be performed in combination with *in vitro* phage display experiments, as previously described by us and other groups.

Over the years*, in vivo* phage display has been proving itself to be an effective and powerful technique not only for the selection of peptides but also for antibody fragments. This is evident in the reviewed studies, where new molecules have been identified for many purposes, such as biomarkers and drug delivery, among many others. Furthermore, with evolving novel and more challenging disease targets and with a new generation of therapeutic antibodies emerging it is clear that *in vivo* phage display will play a key role in antibody development. Thus, with the current advances in phage display techniques, we envision that in the upcoming years many antibodies and peptides will be approved as therapeutic molecules in different areas using the classic *in vitro* phage display technology as well novel *in vivo* phage display methodologies.

## Author contributions

AA: conceptualization, investigation, writing the original draft, review, and editing. IM, JD, and FA-d-S: conceptualization, investigation, writing, review, and editing. All authors contributed to the article and approved the submitted version.

## Funding

This work was supported by the Portuguese Funding Agency, Fundação para a Ciência e Tecnologia, FCT IP (SAICT/2017/32085 and Ph.D. fellowship SFRH/BD/131468/2017 to AA and 2020.08209.BD to IM). CIISA has provided support through Project UIDB/CVT/00276/2020, funded by FCT and LA/P/0059/2020-AL4AnimalS.

## Conflict of interest

The authors declare that the research was conducted in the absence of any commercial or financial relationships that could be construed as a potential conflict of interest.

## Publisher’s note

All claims expressed in this article are solely those of the authors and do not necessarily represent those of their affiliated organizations, or those of the publisher, the editors and the reviewers. Any product that may be evaluated in this article, or claim that may be made by its manufacturer, is not guaranteed or endorsed by the publisher.

## Supplementary material

SUPPLEMENTARY TABLE 1 Approved therapeutic antibody derived from phage display technology.

## References

[ref1] Aguiar S. I. Dias J. N. R. André A. S. Silva M. L. Martins D. Carrapiço B. (2021). Highly specific blood-brain barrier transmigrating single-domain antibodies selected by an in vivo phage display screening. Pharmaceutics 13:1598. doi: 10.3390/pharmaceutics13101598, PMID: 34683891 PMC8540410

[ref2] Aires da Silva F. Corte-Real S. Goncalves J. (2008). Recombinant antibodies as therapeutic agents: pathways for modeling new biodrugs. BioDrugs 22, 301–314. doi: 10.2165/00063030-200822050-00003, PMID: 18778112

[ref3] Aires da Silva F. Santa-Marta M. Freitas-Vieira A. Mascarenhas P. Barahona I. Moniz-Pereira J. (2004). Camelized rabbit-derived VH single-domain intrabodies against Vif strongly neutralize HIV-1 infectivity. J. Mol. Biol. 340, 525–542. doi: 10.1016/j.jmb.2004.04.062, PMID: 15210352

[ref4] Akita N. Maruta F. Seymour L. W. Kerr D. J. Parker A. L. Asai T. (2006). Identification of oligopeptides binding to peritoneal tumors of gastric cancer. Cancer Sci. 97, 1075–1081. doi: 10.1111/j.1349-7006.2006.00291.x, PMID: 16984380 PMC11158424

[ref5] Alfaleh M. A. Alsaab H. O. Mahmoud A. B. Alkayyal A. A. Jones M. L. Mahler S. M. (2020). Phage display derived monoclonal antibodies: from bench to bedside. Front. Immunol. 11:1986. doi: 10.3389/fimmu.2020.01986, PMID: 32983137 PMC7485114

[ref6] Arap W. Haedicke W. Bernasconi M. Kain R. Rajotte D. Krajewski S. (2002a). Targeting the prostate for destruction through a vascular address. Proc. Natl. Acad. Sci. U. S. A. 99, 1527–1531. doi: 10.1073/pnas.241655998, PMID: 11830668 PMC122224

[ref7] Arap W. Kolonin M. G. Trepel M. Lahdenranta J. Cardó-Vila M. Giordano R. J. (2002b). Steps toward mapping the human vasculature by phage display. Nat. Med. 8, 121–127. doi: 10.1038/nm0202-121, PMID: 11821895

[ref8] Bábíčková J. Tóthová Ľ. Boor P. Celec P. (2013). *In vivo* phage display — a discovery tool in molecular biomedicine. Biotechnol. Adv. 31, 1247–1259. doi: 10.1016/j.biotechadv.2013.04.004, PMID: 23623852

[ref9] Barbas C. F. Kang A. S. Lerner R. A. Benkovic S. J. (1991). Assembly of combinatorial antibody libraries on phage surfaces: the gene III site. Proc. Natl. Acad. Sci. U. S. A. 88, 7978–7982. doi: 10.1073/pnas.88.18.7978, PMID: 1896445 PMC52428

[ref10] Bradbury A. R. M. Dübel S. Knappik A. Plückthun A. (2021). Animal-versus in vitro-derived antibodies: avoiding the extremes. MAbs 13:1950265. doi: 10.1080/19420862.2021.1950265, PMID: 34281490 PMC8293942

[ref11] Bradbury A. R. M. Marks J. D. (2004). Antibodies from phage antibody libraries. J. Immunol. Methods 290, 29–49. doi: 10.1016/j.jim.2004.04.007 15261570

[ref010] Barderas R. Benito-Peña E. (2019). The 2018 Nobel Prize in Chemistry: phage display of peptides and antibodies. Anal. Bioanal. Chem. 411, 2475–2479. doi: 10.1007/s00216-019-01714-4 30888467

[ref12] Cantante C. Lourenço S. Morais M. Leandro J. Gano L. Silva N. (2017). Albumin-binding domain from streptococcus zooepidemicus protein Zag as a novel strategy to improve the half-life of therapeutic proteins. J. Biotechnol. 253, 23–33. doi: 10.1016/j.jbiotec.2017.05.017, PMID: 28549690

[ref13] Casey J. King D. Chaplin L. Haines A. Pedley R. Mountain A. (1996). Preparation, characterisation and tumour targeting of cross-linked divalent and trivalent anti-tumour fab’ fragments. Br. J. Cancer 74, 1397–1405. doi: 10.1038/bjc.1996.555, PMID: 8912535 PMC2074792

[ref14] Casey J. L. Napier M. P. King D. J. Pedley R. B. Chaplin L. C. Weir N. (2002). Tumour targeting of humanised cross-linked divalent-fab′ antibody fragments: a clinical phase I/II study. Br. J. Cancer 86, 1401–1410. doi: 10.1038/sj.bjc.6600198, PMID: 11986771 PMC2375360

[ref15] Chi X. Li Y. Qiu X. (2020). V (D) J recombination, somatic hypermutation and class switch recombination of immunoglobulins: mechanism and regulation. Immunology 160, 233–247. doi: 10.1111/imm.13176, PMID: 32031242 PMC7341547

[ref16] de Haard H. J. van Neer N. Reurs A. Hufton S. E. Roovers R. C. Henderikx P. (1999). A large non-immunized human fab fragment phage library that permits rapid isolation and kinetic analysis of high affinity antibodies*. J. Biol. Chem. 274, 18218–18230. doi: 10.1074/jbc.274.26.18218, PMID: 10373423

[ref17] Dennis M. S. Zhang M. Meng Y. G. Kadkhodayan M. Kirchhofer D. Combs D. (2002). Albumin binding as a general strategy for improving the pharmacokinetics of proteins *. J. Biol. Chem. 277, 35035–35043. doi: 10.1074/jbc.M205854200, PMID: 12119302

[ref18] Deramchia K. Jacobin-Valat M.-J. Laroche-Traineau J. Bonetto S. Sanchez S. Dos Santos P. (2012a). By-passing large screening experiments using sequencing as a tool to identify sc Fv fragments targeting atherosclerotic lesions in a novel in vivo phage display selection. IJMS 13, 6902–6923. doi: 10.3390/ijms13066902, PMID: 22837671 PMC3397503

[ref19] Deramchia K. Jacobin-Valat M.-J. Vallet A. Bazin H. Santarelli X. Sanchez S. (2012b). *In vivo* phage display to identify new human antibody fragments homing to atherosclerotic endothelial and subendothelial tissues [corrected]. Am. J. Pathol. 180, 2576–2589. doi: 10.1016/j.ajpath.2012.02.013, PMID: 22521648

[ref20] Dominik P. K. Kossiakoff A. A. (2015). “Chapter eleven-phage display selections for affinity reagents to membrane proteins in Nanodiscs,” in Methods in Enzymology Membrane Proteins—Engineering, Purification and Crystallization. ed. Shukla A. K. (Cambridge, Massachusetts: Academic Press), 219–245. 10.1016/bs.mie.2014.12.032 25950967

[ref21] Duerr D. M. White S. J. Schluesener H. J. (2004). Identification of peptide sequences that induce the transport of phage across the gastrointestinal mucosal barrier. J. Virol. Methods 116, 177–180. doi: 10.1016/j.jviromet.2003.11.012, PMID: 14738985

[ref09] Emmons C. Hunsicker L. G. (1987). Muromonab-CD3 (Orthoclone OKT3): the first monoclonal antibody approved for therapeutic use. Iowa Med 77, 78–82. 3557906

[ref22] Essler M. Ruoslahti E. (2002). Molecular specialization of breast vasculature: a breast-homing phage-displayed peptide binds to aminopeptidase P in breast vasculature. Proc. Natl. Acad. Sci. U. S. A. 99, 2252–2257. doi: 10.1073/pnas.251687998, PMID: 11854520 PMC122351

[ref23] Gray A. Bradbury A. R. M. Knappik A. Plückthun A. Borrebaeck C. A. K. Dübel S. (2020a). Animal-free alternatives and the antibody iceberg. Nat. Biotechnol. 38, 1234–1239. doi: 10.1038/s41587-020-0687-9, PMID: 33046876

[ref24] Gray A. C. Bradbury A. R. M. Knappik A. Plückthun A. Borrebaeck C. A. K. Dübel S. (2020b). Animal-derived-antibody generation faces strict reform in accordance with European Union policy on animal use. Nat. Methods 17, 755–756. doi: 10.1038/s41592-020-0906-9, PMID: 32719533

[ref25] Greenberg A. S. Avila D. Hughes M. Hughes A. McKinney E. C. Flajnik M. F. (1995). A new antigen receptor gene family that undergoes rearrangement and extensive somatic diversification in sharks. Nature 374, 168–173. doi: 10.1038/374168a0, PMID: 7877689

[ref26] Greenwood J. Willis A. E. Perham R. N. (1991). Multiple display of foreign peptides on a filamentous bacteriophage. J. Mol. Biol. 220, 821–827. doi: 10.1016/0022-2836(91)90354-9, PMID: 1880799

[ref27] Griffiths A. D. Duncan A. R. (1998). Strategies for selection of antibodies by phage display. Curr. Opin. Biotechnol. 9, 102–108. doi: 10.1016/S0958-1669(98)80092-X 9503596

[ref28] Griffiths A. D. Williams S. C. Hartley O. Tomlinson I. M. Crosbyl W. L. Jones P. T. (1994). Isolation of high affinity human antibodies directly from large synthetic repertoires. EMBO J. 16, 3245–3260. doi: 10.1002/j.1460-2075.1994.tb06626.x PMC395221 8045255

[ref29] Groves M. Lane S. Douthwaite J. Lowne D. Gareth Rees D. Edwards B. (2006). Affinity maturation of phage display antibody populations using ribosome display. J. Immunol. Methods 313, 129–139. doi: 10.1016/J.JIM.2006.04.002, PMID: 16730741

[ref30] Hamers-Casterman C. Atarhouch T. Muyldermans S. Robinson G. Hammers C. Songa E. B. (1993). Naturally occurring antibodies devoid of light chains. Nature 363, 446–448. doi: 10.1038/363446a0, PMID: 8502296

[ref31] Hamzeh-Mivehroud M. Mahmoudpour A. Rezazadeh H. Dastmalchi S. (2008). Non-specific translocation of peptide-displaying bacteriophage particles across the gastrointestinal barrier. Eur. J. Pharm. Biopharm. 70, 577–581. doi: 10.1016/j.ejpb.2008.06.005, PMID: 18602466

[ref32] Hemadou A. Fontayne A. Laroche-Traineau J. Ottones F. Mondon P. Claverol S. (2021). In vivo human single-chain fragment variable phage display-assisted identification of Galectin-3 as a new biomarker of atherosclerosis. JAHA 10:e016287. doi: 10.1161/JAHA.120.016287, PMID: 34569248 PMC8649142

[ref33] Hemadou A. Laroche-Traineau J. Antoine S. Mondon P. Fontayne A. Le Priol Y. (2018). An innovative flow cytometry method to screen human sc Fv-phages selected by *in vivo* phage-display in an animal model of atherosclerosis. Sci. Rep. 8:15016. doi: 10.1038/s41598-018-33382-2, PMID: 30302027 PMC6177473

[ref34] Holliger P. Hudson P. J. (2005). Engineered antibody fragments and the rise of single domains. Nat. Biotechnol. 23, 1126–1136. doi: 10.1038/nbt1142, PMID: 16151406

[ref35] Holt L. J. Herring C. Jespers L. S. Woolven B. P. Tomlinson I. M. (2003). Domain antibodies: proteins for therapy. Trends Biotechnol. 21, 484–490. doi: 10.1016/j.tibtech.2003.08.007 14573361

[ref36] Hoogenboom H. (1997). Designing and optimizing library selection strategies for generating high-affinity antibodies. Trends Biotechnol. 15, 62–70. doi: 10.1016/S0167-7799(97)84205-9, PMID: 9081300

[ref37] Hoogenboom H. R. (2002). “Overview of antibody phage-display technology and its applications,” in Antibody phage display: Methods and protocols. eds. Brien P. M. O. Aitken R. (Totowa, NJ: Humana Press), 1–37. 10.1385/1-59259-240-6:001 11968478

[ref38] Hoogenboom H. R. (2005). Selecting and screening recombinant antibody libraries. Nat. Biotechnol. 23, 1105–1116. doi: 10.1038/nbt1126, PMID: 16151404

[ref39] Hudson P. J. Souriau C. (2003). Engineered antibodies. Nat. Med. 9, 129–134. doi: 10.1038/nm0103-129 12514726

[ref40] Huston J. S. McCartney J. Tai M. S. Mottola-Hartshorn C. Jin D. Warren F. (1993). Medical applications of single-chain antibodies. Int. Rev. Immunol. 10, 195–217. doi: 10.3109/08830189309061696 8360586

[ref41] Hwang W. Y. K. Foote J. (2005). Immunogenicity of engineered antibodies. Methods 36, 3–10. doi: 10.1016/j.ymeth.2005.01.001 15848070

[ref42] Johns M. George A. J. T. Ritter M. A. (2000). *In vivo* selection of sFv from phage display libraries. J. Immunol. Methods 239, 137–151. doi: 10.1016/S0022-1759(00)00152-6, PMID: 10821955

[ref43] Jones M. L. Alfaleh M. A. Kumble S. Zhang S. Osborne G. W. Yeh M. (2016). Targeting membrane proteins for antibody discovery using phage display. Sci. Rep. 6:26240. doi: 10.1038/srep26240, PMID: 27189586 PMC4870581

[ref44] Khazaeli M. B. Conry R. M. LoBuglio A. F. (1994). Human immune response to monoclonal antibodies. J. Immunother. Emphasis Tumor Immunol. 15, 42–52. doi: 10.1097/00002371-199401000-00006, PMID: 8110730

[ref45] Kirsch M. I. Hülseweh B. Nacke C. Rülker T. Schirrmann T. Marschall H.-J. (2008). Development of human antibody fragments using antibody phage display for the detection and diagnosis of Venezuelan equine encephalitis virus (VEEV). BMC Biotechnol. 8:66. doi: 10.1186/1472-6750-8-66, PMID: 18764933 PMC2543005

[ref46] Knappik A. Ge L. Honegger A. Pack P. Fischer M. Wellnhofer G. (2000). Fully synthetic human combinatorial antibody libraries (HuCAL) based on modular consensus frameworks and CDRs randomized with trinucleotides. J. Mol. Biol. 296, 57–86. doi: 10.1006/JMBI.1999.3444, PMID: 10656818

[ref47] Köhler G. Milstein C. (1975). Continuous cultures of fused cells secreting antibody of predefined specificity. Nature 256, 495–497. doi: 10.1038/256495a0, PMID: 1172191

[ref48] Kolonin M. G. Sun J. Do K. Vidal C. I. Ji Y. Baggerly K. A. (2006). Synchronous selection of homing peptides for multiple tissues by *in vivo* phage display. FASEB J. 20, 979–981. doi: 10.1096/fj.05-5186fje, PMID: 16581960

[ref49] Kontermann R. E. (2011). Strategies for extended serum half-life of protein therapeutics. Curr. Opin. Biotechnol. 22, 868–876. doi: 10.1016/j.copbio.2011.06.012, PMID: 21862310

[ref50] Krag D. N. Shukla G. S. Shen G.-P. Pero S. Ashikaga T. Fuller S. (2006). Selection of tumor-binding ligands in cancer patients with phage display libraries. Cancer Res. 66, 7724–7733. doi: 10.1158/0008-5472.CAN-05-4441, PMID: 16885375

[ref51] Kristensen P. Winter G. (1998). Proteolytic selection for protein folding using filamentous bacteriophages. Fold. Des. 3, 321–328. doi: 10.1016/S1359-0278(98)00044-3, PMID: 9806934

[ref52] Kügler J. Tomszak F. Frenzel A. Hust M. (2018). “Construction of human immune and naive sc Fv libraries,” in Phage display: Methods and protocols. eds. Hust M. Lim T. S. (New York, NY: Springer New York), 3–24.

[ref53] Kügler J. Wilke S. Meier D. Tomszak F. Frenzel A. Schirrmann T. (2015). Generation and analysis of the improved human HAL9/10 antibody phage display libraries. BMC Biotechnol. 15, 1–15. doi: 10.1186/S12896-015-0125-0 25888378 PMC4352240

[ref54] Laakkonen P. Åkerman M. E. Biliran H. Yang M. Ferrer F. Karpanen T. (2004). Antitumor activity of a homing peptide that targets tumor lymphatics and tumor cells. Proc. Natl. Acad. Sci. U. S. A. 101, 9381–9386. doi: 10.1073/pnas.0403317101, PMID: 15197262 PMC438985

[ref55] Lipes B. D. Chen Y.-H. Ma H. Staats H. F. Kenan D. J. Gunn M. D. (2008). An entirely cell-based system to generate single-chain antibodies against cell surface receptors. J. Mol. Biol. 379, 261–272. doi: 10.1016/j.jmb.2008.03.072, PMID: 18455737 PMC2496992

[ref56] Lloyd C. Lowe D. Edwards B. Welsh F. Dilks T. Hardman C. (2009). Modelling the human immune response: performance of a 1011 human antibody repertoire against a broad panel of therapeutically relevant antigens. Protein Engineer. Design Select. 22, 159–168. doi: 10.1093/PROTEIN/GZN058, PMID: 18974080

[ref57] Ma C. Yin G. Yan D. He X. Zhang L. Wei Y. (2013). A novel peptide specifically targeting ovarian cancer identified by *in vivo* phage display: an ovarian cancer-targeting peptide identified by *in vivo* phage display. J. Pept. Sci. 19, 730–736. doi: 10.1002/psc.2555, PMID: 24105738

[ref58] Marks J. D. Hoogenboom H. R. Bonnert T. P. McCafferty J. Griffiths A. D. Winter G. (1991). By-passing immunization. J. Mol. Biol. 222, 581–597. doi: 10.1016/0022-2836(91)90498-U 1748994

[ref59] Maynard J. Georgiou G. (2000). Antibody Engineering. Annual Review of Biomedical Engineering 2. *Vol. 2.* (Palo Alto, California, US: Annual Review), 339–376. 10.1146/annurev.bioeng.2.1.339 11701516

[ref60] McCafferty J. Griffiths A. D. Winter G. Chiswell D. J. (1990). Phage antibodies: filamentous phage displaying antibody variable domains. Nature 348, 552–554. doi: 10.1038/348552a0, PMID: 2247164

[ref61] Molenaar T. J. M. Michon I. de Haas S. A. M. van Berkel T. J. C. Kuiper J. Biessen E. A. L. (2002). Uptake and processing of modified bacteriophage M13 in mice: implications for phage display. Virology 293, 182–191. doi: 10.1006/viro.2001.1254, PMID: 11853411

[ref62] Morrison C. (2019). Nanobody approval gives domain antibodies a boost. Nat. Rev. Drug Discov. 18, 485–487. doi: 10.1038/D41573-019-00104-W, PMID: 31267082

[ref63] Muyldermans S. Cambillau C. Wyns L. (2001). Recognition of antigens by single-domain antibody fragments: the superfluous luxury of paired domains. Trends Biochem. Sci. 26, 230–235. doi: 10.1016/s0968-0004(01)01790-x, PMID: 11295555

[ref64] Nissim A. Hoogenboom H. R. Tomlinson I. M. Flynn G. Midgley C. Lane D. (1994). Antibody fragments from a ‘single pot’ phage display library as immunochemical reagents. EMBO J. 13, 692–698. doi: 10.1002/j.1460-2075.1994.tb06308.x, PMID: 7508862 PMC394860

[ref65] Pande J. Szewczyk M. M. Grover A. K. (2010). Phage display: concept, innovations, applications and future. Biotechnol. Adv. 28, 849–858. doi: 10.1016/j.biotechadv.2010.07.004, PMID: 20659548

[ref66] Parray H. A. Shukla S. Samal S. Shrivastava T. Ahmed S. Sharma C. (2020). Hybridoma technology a versatile method for isolation of monoclonal antibodies, its applicability across species, limitations, advancement and future perspectives. Int. Immunopharmacol. 85:106639. doi: 10.1016/j.intimp.2020.106639, PMID: 32473573 PMC7255167

[ref67] Pasqualini R. Ruoslahti E. (1996). Organ targeting *in vivo* using phage display peptide libraries. Nature 380, 364–366. 8598934 10.1038/380364a0

[ref68] Prassler J. Thiel S. Pracht C. Polzer A. Peters S. Bauer M. (2011). HuCAL PLATINUM, a synthetic fab library optimized for sequence diversity and superior performance in mammalian expression systems. J. Mol. Biol. 413, 261–278. doi: 10.1016/J.JMB.2011.08.012, PMID: 21856311

[ref69] Presta L. G. (2006). Engineering of therapeutic antibodies to minimize immunogenicity and optimize function. Adv. Drug Deliv. Rev. 58, 640–656. doi: 10.1016/j.addr.2006.01.026, PMID: 16904789

[ref70] Reff M. E. Carner K. Chambers K. S. Chinn P. C. Leonard J. E. Raab R. (1994). Depletion of B cells *in vivo* by a chimeric mouse human monoclonal antibody to CD20. Blood 83, 435–445. doi: 10.1182/blood.V83.2.435.435, PMID: 7506951

[ref71] Robert R. Jacobin-Valat M.-J. Daret D. Miraux S. Nurden A. T. Franconi J.-M. (2006). Identification of human sc Fvs targeting atherosclerotic lesions. J. Biol. Chem. 281, 40135–40143. doi: 10.1074/jbc.M609344200, PMID: 17068330

[ref72] Rondot S. Koch J. Breitling F. Dübel S. (2001). A helper phage to improve single-chain antibody presentation in phage display. Nat. Biotechnol. 19, 75–78. doi: 10.1038/83567, PMID: 11135557

[ref73] Rothe C. Urlinger S. Löhning C. Prassler J. Stark Y. Jäger U. (2008). The human combinatorial antibody library HuCAL GOLD combines diversification of all six CDRs according to the natural immune system with a novel display method for efficient selection of high-affinity antibodies. J. Mol. Biol. 376, 1182–1200. doi: 10.1016/J.JMB.2007.12.018, PMID: 18191144

[ref74] Schwimmer L. J. Huang B. Giang H. Cotter R. L. Chemla-Vogel D. S. Dy F. V. (2013). Discovery of diverse and functional antibodies from large human repertoire antibody libraries. J. Immunol. Methods 391, 60–71. doi: 10.1016/J.JIM.2013.02.010, PMID: 23454004

[ref75] Shi L. Wheeler J. C. Sweet R. W. Lu J. Luo J. Tornetta M. (2010). De novo selection of high-affinity antibodies from synthetic fab libraries displayed on phage as pIX fusion proteins. J. Mol. Biol. 397, 385–396. doi: 10.1016/J.JMB.2010.01.034, PMID: 20114051

[ref76] Shim H. (2017). “Antibody phage display,” in *Recombinant antibodies for infectious diseases* advances in experimental medicine and biology. ed. Lim T. S. (Cham: Springer International Publishing), 21–34. 10.1007/978-3-319-72077-7_2 29549633

[ref77] Sievers E. L. Senter P. D. (2013). Antibody-drug conjugates in cancer therapy. Annu. Rev. Med. 64, 15–29. doi: 10.1146/annurev-med-050311-201823 23043493

[ref78] Silva F. Andre A. Dias J. Aguiar S. Oliveira S. Tavares L. (2021). Highly specific rabbit single- domain antibodies for drug delivery in immunotherapy applications. PCT/IB2022/056303.

[ref79] Sleep D. Cameron J. Evans L. R. (2013). Albumin as a versatile platform for drug half-life extension. Biochim. Biophys. Acta Gen. Subj. 1830, 5526–5534. doi: 10.1016/j.bbagen.2013.04.023, PMID: 23639804

[ref80] Smith G. P. (1985). Filamentous fusion phage: novel expression vectors that display cloned antigens on the Virion surface. Science 228, 1315–1317. doi: 10.1126/science.4001944, PMID: 4001944

[ref81] Smith G. P. Petrenko V. A. (1997). Phage display. Chem. Rev. 97, 391–410. doi: 10.1021/cr960065d 11848876

[ref82] Soendergaard M. Newton-Northup J. R. Deutscher S. L. (2014). *In vivo* phage display selection of an ovarian cancer targeting peptide for SPECT/CT imaging. Am. J. Nucl. Med. Mol. Imaging 4, 561–570. 25250205 PMC4171842

[ref83] Spiess C. Zhai Q. Carter P. J. (2015). Alternative molecular formats and therapeutic applications for bispecific antibodies. Mol. Immunol. 67, 95–106. doi: 10.1016/J.MOLIMM.2015.01.003, PMID: 25637431

[ref84] Srivastava A. S. Kaido T. Carrier E. (2004). Immunological factors that affect the *in vivo* fate of T7 phage in the mouse. J. Virol. Methods 115, 99–104. doi: 10.1016/j.jviromet.2003.09.009, PMID: 14656466

[ref85] Staquicini F. I. Cardó-Vila M. Kolonin M. G. Trepel M. Edwards J. K. Nunes D. N. (2011). Vascular ligand-receptor mapping by direct combinatorial selection in cancer patients. Proc. Natl. Acad. Sci. U. S. A. 108, 18637–18642. doi: 10.1073/pnas.1114503108, PMID: 22049339 PMC3219136

[ref86] Stark Y. Venet S. Schmid A. (2017). “Whole cell panning with phage display,” in *Synthetic antibodies: Methods and protocols* methods in molecular biology. ed. Tiller T. (New York, NY: Springer), 67–91. 10.1007/978-1-4939-6857-2_5 28255875

[ref87] Sterner R. C. Sterner R. M. (2021). CAR-T cell therapy: current limitations and potential strategies. Blood Cancer J. 11, 69–11. doi: 10.1038/s41408-021-00459-7, PMID: 33824268 PMC8024391

[ref88] Stork R. Muller D. Kontermann R. E. (2007). A novel tri-functional antibody fusion protein with improved pharmacokinetic properties generated by fusing a bispecific single-chain diabody with an albumin-binding domain from streptococcal protein G. Protein Eng. Des. Sel. 20, 569–576. doi: 10.1093/protein/gzm061, PMID: 17982179

[ref89] Stutz C. C. Georgieva J. V. Shusta E. V. (2018). Coupling brain perfusion screens and next generation sequencing to identify blood–brain barrier binding antibodies. AICHE J. 64, 4229–4236. doi: 10.1002/aic.16360, PMID: 30872841 PMC6411078

[ref90] Tan Y. Tian T. Liu W. Zhu Z. Yang J. (2016). Advance in phage display technology for bioanalysis. Biotechnol. J. 11, 732–745. doi: 10.1002/biot.201500458, PMID: 27061133

[ref91] Ueberberg S. Meier J. J. Waengler C. Schechinger W. Dietrich J. W. Tannapfel A. (2009). Generation of novel single-chain antibodies by phage-display technology to direct imaging agents highly selective to pancreatic β-or α-cells in vivo. Diabetes 58, 2324–2334. doi: 10.2337/db09-0658, PMID: 19592622 PMC2750237

[ref92] Ueberberg S. Schneider S. (2010). Phage library-screening: a powerful approach for generation of targeting-agents specific for normal pancreatic islet-cells and islet-cell carcinoma *in vivo*. Regul. Pept. 160, 1–8. doi: 10.1016/j.regpep.2009.11.017, PMID: 19958795

[ref93] Valadon P. Pérez-Tapia S. M. Nelson R. S. Guzmán-Bringas O. U. Arrieta-Oliva H. I. Gómez-Castellano K. M. (2019). ALTHEA Gold libraries™: antibody libraries for therapeutic antibody discovery. MAbs 11, 516–531. doi: 10.1080/19420862.2019.1571879, PMID: 30663541 PMC6512909

[ref94] van Lith S. A. M. Roodink I. Verhoeff J. J. C. Mäkinen P. I. Lappalainen J. P. Ylä-Herttuala S. (2016). *In vivo* phage display screening for tumor vascular targets in glioblastoma identifies a llama nanobody against dynactin-1-p150Glued. Oncotarget 7, 71594–71607. doi: 10.18632/oncotarget.12261, PMID: 27689404 PMC5342104

[ref95] Vaughan T. J. Williams A. J. Pritchard K. Osbourn J. K. Pope A. R. Earnshaw J. C. (1996). Human antibodies with sub-nanomolar affinities isolated from a large non-immunized phage display library. Nat. Biotechnol. 14, 309–314. doi: 10.1038/NBT0396-309, PMID: 9630891

[ref96] Veleva A. N. Nepal D. B. Frederick C. B. Schwab J. Lockyer P. Yuan H. (2011). Efficient in vivo selection of a novel tumor-associated peptide from a phage display library. Molecules 16, 900–914. doi: 10.3390/molecules16010900, PMID: 21258297 PMC3328215

[ref97] Wang L. Wang N. Zhang W. Cheng X. Yan Z. Shao G. (2022). Therapeutic peptides: current applications and future directions. Sig. Transduct. Target Ther. 7:48. doi: 10.1038/s41392-022-00904-4, PMID: 35165272 PMC8844085

[ref98] Ward R. L. Clark M. A. Lees J. Hawkins N. J. (1996). Retrieval of human antibodies from phage-display libraries using enzymatic cleavage. J. Immunol. Methods 189, 73–82. doi: 10.1016/0022-1759(95)00231-6, PMID: 8576582

[ref99] Weber M. Bujak E. Putelli A. Villa A. Matasci M. Gualandi L. (2014). A highly functional synthetic phage display library containing over 40 billion human antibody clones. PLoS One 9:e100000. doi: 10.1371/JOURNAL.PONE.0100000, PMID: 24950200 PMC4065035

[ref100] Weir A. N. C. Nesbitt A. Chapman A. P. Popplewell A. G. Antoniw P. Lawson A. D. G. (2002). Formatting antibody fragments to mediate specific therapeutic functions. Biochem. Soc. Trans. 30, 512–516. doi: 10.1042/bst0300512, PMID: 12196125

[ref101] Whitney M. Crisp J. L. Olson E. S. Aguilera T. A. Gross L. A. Ellies L. G. (2010). Parallel in vivo and in vitro selection using phage display identifies protease-dependent tumor-targeting peptides. J. Biol. Chem. 285, 22532–22541. doi: 10.1074/jbc.M110.138297, PMID: 20460372 PMC2903386

[ref102] Williams A. F. Barclay A. N. (1988). The immunoglobulin superfamily--domains for cell surface recognition. Annu. Rev. Immunol. 6, 381–405. doi: 10.1146/annurev.iy.06.040188.002121, PMID: 3289571

[ref103] Winter G. Griffiths A. D. Hawkins R. E. Hoogenboom H. R. (1994). Making antibodies by phage display technology. Annu. Rev. Immunol. 23, 433–455. 10.1146/annurev.iy.12.040194.002245 8011287

[ref104] Wu M. Pasula R. Smith P. A. Martin W. J. (2003). Mapping alveolar binding sites *in vivo* using phage peptide libraries. Gene Ther. 10, 1429–1436. doi: 10.1038/sj.gt.3302009, PMID: 12900757

[ref105] Zou J. Dickerson M. T. Owen N. K. Landon L. A. Deutscher S. L. (2004). Biodistribution of filamentous phage peptide libraries in mice. Mol. Biol. Rep. 31, 121–129. doi: 10.1023/b:mole.0000031459.14448.af, PMID: 15293788

[ref106] Zurita A. J. Troncoso P. Cardó-Vila M. Logothetis C. J. Pasqualini R. Arap W. (2004). Combinatorial screenings in patients: the Interleukin-11 receptor α as a candidate target in the progression of human prostate cancer. Cancer Res. 64, 435–439. doi: 10.1158/0008-5472.CAN-03-2675, PMID: 14744752

