# Supplementary material for: In vivo Phage Display: A promising selection strategy for the improvement of antibody targeting and drug delivery properties
Source: Front Microbiol. 2022 Sep 26;13:962124. doi: 10.3389/fmicb.2022.962124 (PMC9549074; doi:10.3389/fmicb.2022.962124)
Supplement: SUPPLEMENTARY TABLE 1 — Approved therapeutic antibody derived from phage display technology. [file Table_1.docx]

**Supplementary material**

**Table S1** - Approved therapeutic antibody derived from phage display technology.

| **Product/Brand name** | **Antibody format** | **Target** | **Phage Display Library Type** | **Phage Display Technology** | **Clinical Application** | **Indications**  **Approved year** | **Sponsor company** |
| --- | --- | --- | --- | --- | --- | --- | --- |
| Adalimumab/Humira® | IgG1-κ | TNFα | Humanization using guided selection method  scFv library | CAT | Immunology and inflammation  (Pelechas et al., 2021) (Brankov and Jacob, 2022) (Jemec et al., 2019) | RA  Approved 2002 | AbbVie |
|  |  |  |  |  |  | PSA  Approved 2005 |  |
|  |  |  |  |  |  | AS  Approved 2006 |  |
|  |  |  |  |  |  | CD  Approved 2007 |  |
|  |  |  |  |  |  | Psoriasis,  Severe chronic plaque  Approved 2008 |  |
|  |  |  |  |  |  | JIA  Approved 2008 |  |
|  |  |  |  |  |  | UC  Approved 2012 |  |
|  |  |  |  |  |  | HS  Approved 2015 |  |
|  |  |  |  |  |  | Fingernail psoriasis Approved 2017 |  |
|  |  |  |  |  | Ophthalmology  (Suhler et al., 2021) (Burek-Michalska and Turno-Kręcicka, 2020) | Uveitis  Approved 2016 |  |
| Ranibizumab/Lucentis® | Fab-IgG1-κ | VEGFA | In vitro affinity maturation of bevacizumab | Genentech | Immunology and Ophthalmology  (Holekamp et al., 2022) (Prünte et al., 2016) (Toscano et al., 2021) | nAMD  Approved 2006 | Roche/Novartis |
|  |  |  |  |  |  | MEfRVO  Approved 2010 |  |
|  |  |  |  |  |  | DME  Approved 2012 |  |
|  |  |  |  |  |  | CNV  Approved 2016 |  |
|  |  |  |  |  |  | Diabetic retinopathy Approved 2017 |  |
| Belimumab/Benlysta® | IgG1-λ | BLyS | Human naïve scFv library | CAT | Immunology and inflammation  (Stohl et al., 2017) (Brunner et al., 2020) | SLE  Approved 2011 | GlaxoSmithKline/Human Genome Sciences |
| Raxibacumab/ABthrax® | IgG1-λ | Bacillus anthrasis PA | Human naïve scFv library | CAT | Infectious diseases  (Tsai and Morris, 2015) | Inhalation anthrax  Approved 2012 | GlaxoSmithKline/Human Genome Sciences |
| Ramucirumab/Cyramza® | IgG1-κ | VEGFR2 | Human naïve Fab library | Dyax | Oncology  (Mehta et al., 2020) (Tabernero et al., 2015) (Zhu et al., 2019) | Gastric cancer  NSCLC  Approved 2014 | Eli Lilly |
|  |  |  |  |  |  | Colorectal cancer  Approved 2015 |  |
|  |  |  |  |  |  | HCC  Approved 2019 |  |
| Necitumumab/Portrazza® | IgG1-κ | EGFR | Human naïve Fab library | Dyax | Oncology  (Garnock-Jones, 2016)  (Fala, 2016) (Besse et al., 2020) | NSCLC  Approved 2015 | Eli Lilly |
| Ixekizumab/Taltz® | IgG4-κ | IL-17A | Mouse immune Fab library | Eli Lilly | Immunology (Lespessailles and Toumi, 2021) (Blauvelt et al., 2020) (Liu et al., 2016)(Dougados et al., 2020) | Psoriasis  Approved 2016 | Eli Lilly |
|  |  |  |  |  |  | PSA  Approved 2017 |  |
|  |  |  |  |  |  | AS  Approved 2019 |  |
| Atezolizumab/Tecentriq® | IgG1-κ | PD-L1 | Human  naïve library | Genentech | Oncology  (Herbst et al., 2020) (van der Heijden et al., 2021) (Mittendorf et al., 2020) | NSCLC  Approved 2016 | Roche |
|  |  |  |  |  |  | Urothelial Carcinoma Approved 2016 |  |
|  |  |  |  |  |  | Urothelial bladder cancer  Approved 2017 |  |
|  |  |  |  |  |  | Breast cancer  Approved 2019 |  |
| Avelumab/Bavencio® | IgG1-λ | PD-L1 | Human naïve Fab library | Dyax | Oncology  (D’Angelo et al., 2021) (Kim, 2017) (Grivas et al., 2021) (Cathomas et al., 2022) (Apolo et al., 2020) | mMCC  Metastatic urothelial carcinoma  Approved 2017 | Merck Serono International S.A./Pfizer |
|  |  |  |  |  |  | RCC Approved 2019 |  |
| Guselkumab/Tremfya® | IgG1-λ | IL-23 | Synthetic Fab library | Morphosys’s HuCAL GOLD® | Immunology  (Coates et al., 2022) (Reich et al., 2021) | Psoriasis  Approved 2017 | MorphoSys/Janssen Biotech Inc. |
| Caplacizumab/Cablivi® | Humanized V_H_-V_H_ | vWF | Immune camelidae-derived nanobody library | Nanobody® | Cardiology and hematology  (Duggan, 2018) (Knoebl et al., 2020) (Sargentini-Maier et al., 2019) | aTTP  Approved 2018 | Sanofi/Ablynx |
| Emapalumab/Gamifant® | IgG1-λ | IFNγ | Human naïve scFv library | CAT | Hematology  (Al-Salama, 2019) (Triebwasser et al., 2021) | HLH  Approved 2018 | NovImmune SA |
| Moxetumomab pasudotox/Lumoxiti® | Murine IgG1 dsFv and a Pseudomonas exotoxin A | CD22 | *In vitro* affinity maturation | CAT | Oncology  (Robak et al., 2021) (Janus and Robak, 2019) (Abou Dalle and Ravandi, 2019) | HCL  Approved 2018 | MedImmune/AstraZeneca |
| Lanadelumab/Takhzyro® | IgG1-κ | pKal | Human naïve Fab library | Dyax | Immunology  (Riedl et al., 2020) (Banerji et al., 2018) | HAE  Approved 2018 | Dyax Corp/Shire |

Data current as of August 22, 2022.

Abbreviations: TNFα: Tumor necrosis factor-alpha; RA: Rheumatoid arthritis; PSA: Psoriatic arthritis; AS: Ankylosing spondylitis; CD: Crohn's disease; JIA:, Juvenile Idiopathic Arthritis; UC: Ulcerative colitis; HS: Hidradenitis suppurativa; nAMD: Neovascular age-related macular degeneration; MEfRVO: Macular edema following Retinal Vein Occlusion; DME: Diabetic macular edema; CNV, Visual impairment due to choroidal neovascularization; PD-L1: Programmed cell death-ligand 1; NSCL: Non-small cell lung cancer; RCC: Renal cell carcinoma; HCC: Hepatocellular carcinoma; mMCC: metastatic Merkel cell carcinoma; BLyS: B-lymphocyte stimulator; SLE: Systemic Lupus Erythematosus; IL-17A: Interleukin-17A; HCL: Hairy cell leukemia; EGFR: Epidermal Growth Factor Receptor; VEGFR2: Vascular endothelial growth factor receptor 2; VEGFA: Vascular endothelial growth factor A; nAMD: Neovascular age-related macular degeneration; MEfRVO: Macular edema following Retinal Vein Occlusion; DME: Diabetic macular edema; CNV: Visual impairment due to choroidal neovascularization; PA: Protective antigen; pKal: Plasma kallikrelin; HAE: Hereditary Angioedema; HLH: Hemophagocytic lymphohistiocytosis.
